# CRISPR knockout screening identifies combinatorial drug targets in pancreatic cancer and models cellular drug response

**DOI:** 10.1038/s41467-018-06676-2

**Published:** 2018-10-15

**Authors:** Karol Szlachta, Cem Kuscu, Turan Tufan, Sara J. Adair, Stephen Shang, Alex D. Michaels, Matthew G. Mullen, Natasha Lopes Fischer, Jiekun Yang, Limin Liu, Prasad Trivedi, Edward B. Stelow, P. Todd Stukenberg, J. Thomas Parsons, Todd W. Bauer, Mazhar Adli

**Affiliations:** 1 Department of Biochemistry and Molecular Genetics, University of Virginia School of Medicine, 1340 JPA, Pinn Hall, Charlottesville, VA 22908 USA; 2 Department of Surgery, University of Virginia School of Medicine, 1215 Lee St, Charlottesville, VA 22908 USA; 3 Department of Pathology, University of Virginia School of Medicine, Charlottesville, 1215 Lee St, Charlottesville, VA 22908 USA; 4 Department of Microbiology, Immunology, and Cancer Biology, University of Virginia School of Medicine, 1340 JPA, Pinn Hall, Charlottesville, VA 22908 USA

**Keywords:** Cancer, Computational biology and bioinformatics

## Abstract

Predicting the response and identifying additional targets that will improve the efficacy of chemotherapy is a major goal in cancer research. Through large-scale in vivo and in vitro CRISPR knockout screens in pancreatic ductal adenocarcinoma cells, we identified genes whose genetic deletion or pharmacologic inhibition synergistically increase the cytotoxicity of MEK signaling inhibitors. Furthermore, we show that CRISPR viability scores combined with basal gene expression levels could model global cellular responses to the drug treatment. We develop drug response evaluation by in vivo CRISPR screening (DREBIC) method and validated its efficacy using large-scale experimental data from independent experiments. Comparative analyses demonstrate that DREBIC predicts drug response in cancer cells from a wide range of tissues with high accuracy and identifies therapeutic vulnerabilities of cancer-causing mutations to MEK inhibitors in various cancer types.

## Abstract (web summary)

Predicting the response to chemotherapy is a major goal of cancer research. Here the authors use CRISPR knockout screens in pancreatic ductal adenocarcinoma cells to identify deletions synergistic with MEK inhibitors.

## Introduction

Pancreatic ductal adenocarcinoma (PDAC) is one of the deadliest cancer types with a median survival time of 6–12 months^1^. Moreover, the statistics for PDAC have remained nearly unchanged for 50 years^2^, and it is projected to be the second leading cause of cancer death in the United States by 2030^3^. At the genetic level, the major gene mutations and aberrant signaling pathways that drive PDAC are well established^4,5^. Oncogenic *KRAS* mutations are observed in 93% of the patients^4^. Additionally, mutations in *CDKN2A*, *TP53*, and *SMAD4* tumor suppressor genes are highly incident in PDAC. Oncogenic *KRAS* mutations aberrantly activate multiple downstream signaling pathways in PDAC^5^. Among these, the RAS–RAF–MEK–ERK pathway is the major driver of tumor formation by providing survival signals to the cancer cell. This knowledge led the expectations that targeted inhibition of the MEK signaling pathway is a promising therapeutic approach in PDAC and other diseases with aberrant RAS–RAF–MEK signaling^6^. Promising clinical results in melanoma, a disease where this signaling pathway is aberrantly active due to *BRAF* mutations^7^, demonstrated the therapeutic value of targeted inhibition of mitogen-activated protein kinase-1/2 (MEK1/2). Unfortunately, MEK inhibitors alone or combined with gemcitabine did not show promising results in clinical trials for PDAC.

Identifying effective therapeutic combinations and tailoring medical treatments according to the characteristics of an individual is the ultimate goal of cancer research and precision medicine^8^. However, predicting a patient’s cellular response to a drug remains a formidable challenge^9^. This is largely because of our limited understanding of the full spectrum of drug targets, their relative importance for drug response, and their abundance in cells and tumors.

Here, we use a large-scale CRISPR (Clustered Regularly Interspaced Short Palindromic Repeats) genetic knockout (KO) screening approach^10–12^ to identify genes whose depletion will positively or negatively alter the survival of PDAC cells when MEK signaling pathway is inhibited. We perform in vitro and in vivo KO screening in a patient-derived xenograft cell line of PDAC. We identify multiple therapeutically targetable genes whose depletion synergistically increases cellular sensitivity to MEK inhibition. We validate several of the top hits with targeted genetic deletions as well as small molecule inhibitors. We also develop a novel drug response prediction method that integrates the combined actions of drug fitness genes from the CRISPR screen with basal gene expression levels. To validate this DREBIC (drug response evaluation by in vivo CRISPR screening) approach, we utilize experimental drug response data from the Cancer Cell Line Encyclopedia (CCLE)^13,14^ and the Cancer Genome Project (CGP)^15^. Our results show that DREBIC models cellular response to MEK inhibitors with high sensitivity and specificity. Furthermore, mutation-specific DREBIC analysis identifies known and novel genetic alterations that modulate overall cellular fitness to MEK inhibitors. In conclusion, our findings demonstrate that CRISPR screens can be utilized to identify genetic targets of drugs and that the DREBIC-like approaches enable precision medicine by modeling overall drug responses and identifying drug-specific therapeutic vulnerabilities of cancer-causing mutations.

## Results

### Performing large-scale CRISPR KO screening in in vivo

**Fig. 1:**
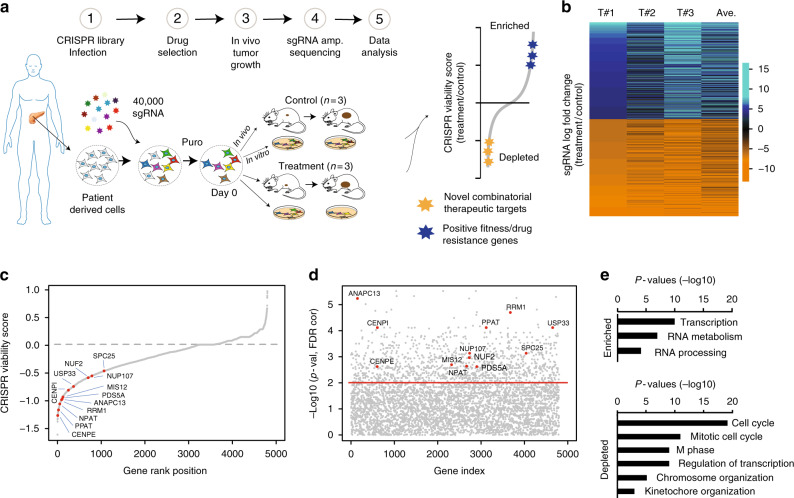
In vivo CRISPR screening identifies novel drug targets. **a** The experimental outline of in vivo genetic knockout screening in PDX model of PDAC. **b** The heat map represents log-fold change of the top 2000 enriched and depleted sgRNAs in three separate trametinib-treated tumors relative to the mean of control-treated tumors. **c** Plot showing the distribution of normalized CRISPR viability scores (treatment vs control) for genes targeted by the sgRNA library. Shown are all targeted genes. Red dots indicate significantly depleted kinetochore genes as assessed by one-sided Kolmogorov–Smirnov test. **d** The plot showing the significance score (treatment vs control) of all gene depletion (minus log-transformed *p* values) as determined by Kolmogorov–Smirnov test. Kinetochore genes are labeled with red dots. **e** Gene ontology (GO) analysis depicts the significantly enriched GO terms for the top enriched and depleted gene sets (*n* = 100) from in vivo screening. Log-transformed *p* values are represented by bar plots

To perform the CRISPR screening schematized in Fig. 1a, we used a clinically relevant patient-derived xenograft (PDX) model of PDAC^16,17^ in which a patient’s tumor is propagated in vivo within the pancreas of athymic nude mice. Due to its efficient vascularization and robust tumor formation capacity, this model retains the biological properties of the original tumor while it grows in the orthotopic microenvironment^16^. We used the PDX366 model which is established from a poorly differentiated metastatic tumor with low stromal content and mutant for *KRAS*, *P53*, and *SMAD4* but wild type (WT) for *P16* genes^18^. In our CRISPR screen, we used “nuclear” single-guide RNA (sgRNA) library targeting ~4000 human genes enriched for epigenetic regulators, transcription factors, and nuclear proteins^11^.

To maintain the sgRNA coverage, we infected ~150–200 million cells at ~0.3 multiplicity of infection (MOI). After a week of drug selection, the surviving cells were randomly divided into 9 batches each containing ~8 million cells (~200× sgRNA coverage). Of these, one sample was harvested as day 0 and others were maintained in culture for in vitro screening or for xenograft injection into the pancreas of athymic nude mice (~8 million cells/mouse, 6 mice total). At 1 week after injection, animals were randomized to receive either vehicle control (*n* = 3) or trametinib treatment (*n* = 3) for 4 weeks as described in the Methods. The relative abundance of each sgRNA was assessed by targeted amplification and deep sequencing of tumor genomic DNA. Data analysis was performed using previously established analytic tools^19^. In parallel, we also performed in vitro screening, in which cultured cells were exposed to control dimethyl sulfoxide (DMSO) or 20% inhibitory concentration (IC_20_) doses of trametinib every 3 days for 4 weeks.

The sgRNA distribution analyses showed that ~94% of the sgRNAs were detectable in control in vitro samples. In contrast, on average, 64% of the sgRNAs were detectable in untreated tumors, suggesting that ~70% of cells containing sgRNAs contributed to in vivo tumor formation (Supplementary Figure 1, Supplementary Table 1, Supplementary Data 1). Notably, a comparative analysis of the overall in vitro and in vivo sgRNA distributions shows that sgRNAs are globally depleted in in vivo tumors, indicating that cellular engraftment is a major barrier for proper representation of sgRNAs in in vivo screens (Supplementary Figure 2). Interestingly, the comparative gene-specific sgRNA enrichment analysis further highlights the global depletion of sgRNA in vivo, but it also shows that sgRNAs targeting certain genes are depleted more profoundly in vivo (Supplementary Figure 3a-b). Notably, genes that play a key role in cell adhesion and migration such as *IL8*^20,21^, cadherin coding *FZR1*^22^, and metastasis-related *EGFL6*^23^ are among the most depleted genes discovered in the in vivo screen (Supplementary Figure 3a). Overall, CRISPR-mediated gene-based viability scores also identified genes that are consistently depleted in both in vitro and in vivo control samples. As expected, these genes are enriched for known essential fitness functions^24^, confirming that the sgRNA depletion in our screening is due to a functional genetic knockout phenotype (Supplementary Figure 4). Despite the in vivo and in vitro differences in overall sgRNA distributions, gene ontology (GO) analysis on the top 100 enriched and depleted genes indicates comparable GO terms and signaling pathways (Supplementary Figure 5).

### Finding conditionally lethal drug targets of MEK inhibition

Notably, mitotic cell cycle and chromosome segregation were the top GO terms for depleted sgRNAs (Supplementary Figure 5). The sgRNA enrichment and depletion analysis across different PDX tumors that were isolated from different mice indicated consistent enrichment scores (Fig. 1b). Gene-based CRISPR viability scores revealed multiple genes that positively or negatively impact the overall cellular fitness to trametinib (Fig. 1c). The enriched sgRNAs in trametinib-treated tumors suggest that KO of their gene targets increased the overall fitness to drug treatment. On the other hand, the depleted sgRNAs suggest that inhibition of their gene targets would make cells significantly more sensitive to trametinib treatment, thus providing a novel target for an effective combinatorial treatment. Notably, the top sgRNAs that are consistently depleted across all tumors are targeting critical mitotic cell cycle and kinetochore function such as *CENPE*, *NUF2*, *MIS12*, and *CENPI* (Fig. 1c, d). Additionally, several other potentially druggable genes that encode targetable enzymes such as the catalytic subunit of ribonucleotide reductase *RRM1* was among the top hits in the screen. Panther gene ontology analysis for the top 100 most consistently depleted genes show significant enrichment of mitotic cell cycle and kinetochore-related gene ontology terms. The enriched genes also indicate involvement in general transcription and RNA metabolism, which are expected terms since our sgRNA pool is targeting transcription factors and nuclear proteins (Fig. 1e).

**Fig. 2:**
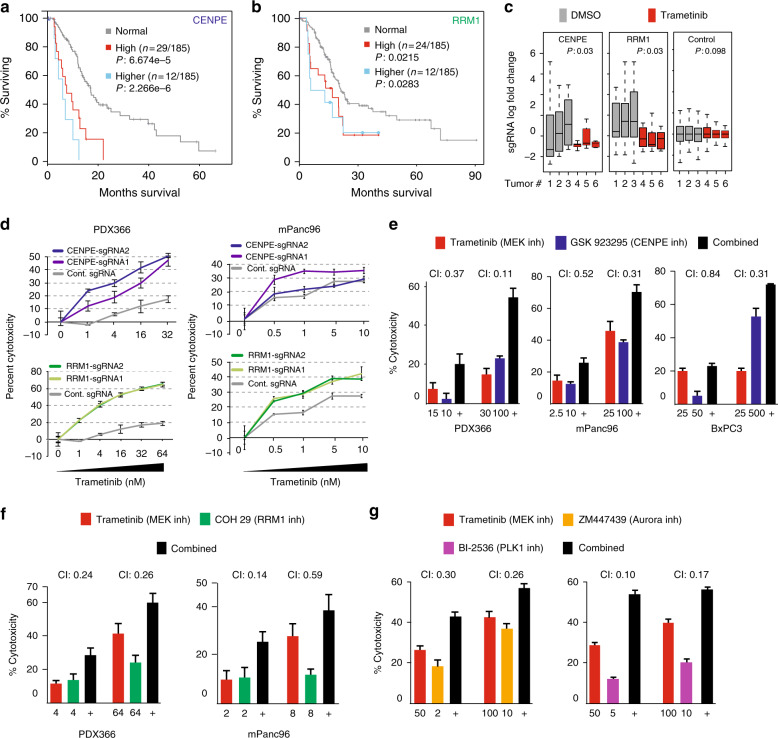
The validation of CRISPR screening hits. **a**, **b** Kaplan–Meier plots showing the survival analysis of TCGA data for pancreatic cancer patients expressing high levels of *CENPE* and *RRM1* genes. High expression: >1 standard deviation (SD), and higher expression: >2 SD above the population mean. The number of patients in each group is indicated with *n*. **c** Box plots show fold change distribution of sgRNAs targeting *CENPE* (*n* = 10), *RRM1* (*n* = 10), and control sgRNAs (*n* = 100) in tumors from control-treated and trametinib-treated mice. Bounds of the box spans from 25 to 75% percentile, center line represents median, and whiskers visualize 5 and 95% of the data points. Significance was assessed by Kolmogorov–Smirnov test. **d** MTT assay-based relative viability/growth inhibition levels are shown in PDX366 and mPanc96 cells expressing WT Cas9 with control sgRNA or two separate sgRNAs targeting *CENPE* (top) and *RRM1* genes (bottom). **e** Relative viability/growth inhibition levels are shown for three different cell lines, PDX366, MPANC-96, and BxPC3 cells, treated with control, MEK inhibitor (trametinib), CENPE inhibitor (GSK923295), and combined MEK inhibitor and CENPE inhibitor. **f** RRM1 inhibitor (COH29) and trametinib were tested together in PDX366 and MPANC-96 cells. **g** Bar plots show relative growth inhibition of MPANC-96 cells treated with inhibitors of Aurora A/B (ZM447439) and PLK1 inhibitor (BI-2536) alone or in combination with trametinib. Each experiment was repeated at least three times and error bars represent the standard error of the mean value. CI cytotoxicity index, Comb combination of two drugs

For validation experiments, we focused our efforts on *CENPE* and *RRM1* because of their biological significance in pancreatic adenocarcinoma and the availability of validated small molecule inhibitors. *RRM1* encodes the catalytic subunit of ribonucleotide reductase. Because of its critical role in cell cycle and DNA synthesis, the enzyme presents itself as a potential therapeutic target in PDAC and other cancers^25^. CENPE is a kinetochore-associated protein that is required for chromosome congression and also for a robust mitotic checkpoint signal transduction^26–28^. Critically, the cancer genome atlas (TCGA) analysis shows that higher expression of both genes is significantly associated with poor prognosis in PDAC (Fig. 2a, b). The relative frequency analysis of sgRNAs targeting *CENPE* or *RRMI* shows that they are specifically depleted in trametinib-treated tumors only (Fig. 2c), thus demonstrating that the depletion of these genes creates a specific conditional lethality upon trametinib treatment. To further confirm these findings, we genetically targeted and depleted these *CENPE* and *RRM1* in PDX366 and mPanc96 cells using CRISPR/Cas9 system (Supplementary Figure 6). As shown in Fig. 2d, cells with sgRNAs targeting *CENPE* and *RRM1* are significantly more sensitive to trametinib treatment compared to control sgRNAs. To further corroborate the genetic depletion results and understand the potential mechanism behind this conditional lethality, we pharmacologically targeted these proteins using specific small molecule inhibitors. To target CENPE, we used GSK923295 allosteric inhibitor^29^ and to target RRM1 we used COH29 small molecule that targets ligand-binding pocket of RRM1^30^ in multiple pancreatic cancer cell lines. Critically, we observed strong synergy between MEK and CENPE inhibition as well as MEK and RRM1 inhibition in three different pancreatic cancer cells at several different drug concentrations by measuring combination index (CI <1)^31,32^ (Fig. 2e, f).

Since multiple other kinetochore genes were among our top hits, we set to test the hypothesis that impaired kinetochore function creates conditional lethality with MEK inhibition. During mitosis, cells are dependent upon a robust spindle assembly checkpoint (SAC) to arrest anaphase onset until each chromosome is aligned to the metaphase^33^. They are also dependent upon an anti-apoptotic signal to prevent death during the prolonged mitosis^34^. When the SAC is compromised, cells undergo mitotic slippage when they exit mitosis before the chromosomes align^35,36^. When the anti-apopotic signal is compromised, cells die during mitosis. Since CENPE depletion results in unaligned chromosomes and a compromised checkpoint^37,38^, we hypothesized that MEK inhibition either further compromises the spindle checkpoint or generates cell death during mitosis. To test this, we combined MEK inhibitors with small molecule inhibitors of Aurora A/B and polo-like kinase-1 (PLK1) kinases that are also required for robust SAC signaling and kinetochore function^39–42^. Importantly, although we observed positive synergy with inhibitors of Aurora A/B (ZM447439), the strongest synergy was observed for PLK1 inhibitor (BI-2536) (CI = 0.1 vs 0.3) (Fig. 2g), suggesting that MEK signaling and the PLK1 pathway play redundant roles in spindle function. However, this experiment cannot distinguish whether MEK inhibition compromises the SAC or whether MEK is required to generate the anti-apoptotic signal in mitosis.

### MEK is required for prolonged mitotic arrest

**Fig. 3:**
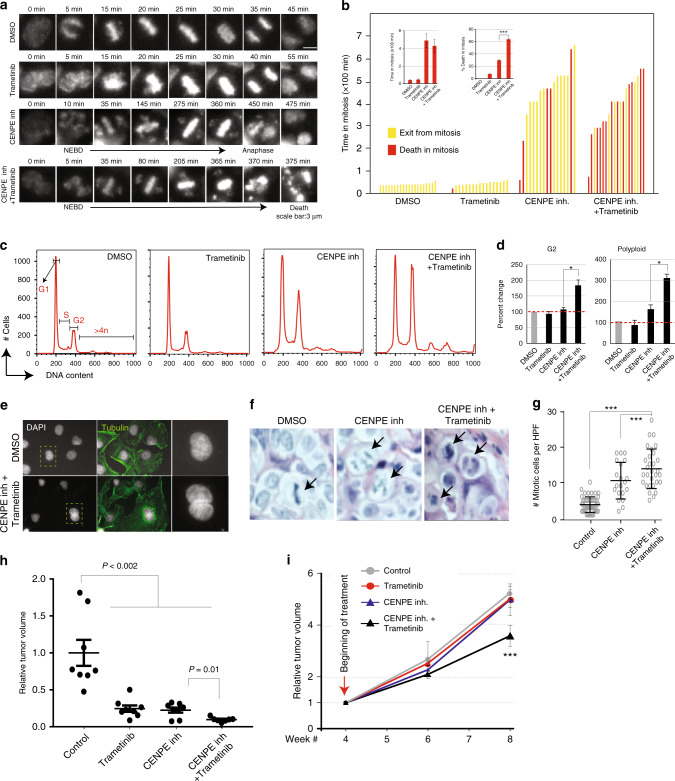
Combinatorial MEK and CENPE inhibition results in synergistic cell death. **a** Each frame represents movie stills from time-lapse longer-term live-cell imaging as cells undergo mitosis starting from nuclear envelope break down (NEBD) to exit from mitosis. **b** Each bar graph show total time duration of each of the individually tracked cells spent in mitosis. The NEBD was taken as the beginning of mitosis. Individual cells (*n* = ~20/treatment group) were manually tracked from lime-lapse movies until they exit mitosis (anaphase) or died in mitosis for each of the treatment groups. **c** Flow cytometry profiles of DNA content in PDX366 PDAC cells treated with control, single-agent, or combinatorial MEK and CENPE inhibitors are shown. **d** Bar graphs represent treatment-mediated percent changes in cells with 2n (G1), 4n (G2), and >4n (polyploidy) DNA. **e** Immunofluorescent images of PDX366 PDAC cells treated with control and combinatorial (trametinib and CENPE inhibitors) are shown after DAPI and tubulin staining in the first two left columns respectively. **f** Representative hematoxylin and eosin (H&E)-stained tumor sections are shown. Tumors were harvested from mice that had undergone 72 h of treatment with 125 mg/kg CENPE inhibitor alone or in combination with 0.3 mg/kg trametinib. Arrows indicate mitotic figures. **g** Dot-plot showing the number of mitotic cells quantified from 10 different high-power imaging fields (HPFs) per tumor section. **h** Results show MRI-measured effects of single and combination of drugs on tumor formation. At 1 week after orthotopic implant, mice received control, MEK inhibitor (trametinib), CENPE inhibitor (GSK923295), or combined MEK and CENPE inhibitor. Results show MRI-measured tumor volumes after 4 weeks of treatment. **i** MRI-measured tumor volumes are shown in mice where tumors were allowed forming for 4 weeks and the treatments (as in **e**) were started. Beginning of treatment is marked with an arrow. Bounds of the box spans from 25 to 75% percentile, center line represents median, and whiskers visualize 5 and 95% of the data points. Individual data points are marked with circles or dots. Error bars represent the standard error of the mean value of at least three independent experiments. Symbols * and *** denote *P* < 0.05 and *P* < 0.001, respectively

To directly test the role of MEK in generating a robust SAC in CENPE-depleted cells, we utilized long-term live imaging approach to measure the timing of cell death (Fig. 3a). Specifically, we treated cells with single or combinatorial drugs and took >24 h movies to track mitotic behaviors of individual cells under various treatment conditions. Notably, control cells complete the mitotic process (from nuclear membrane break down to anaphase) in ~45 min and trametinib treatment did not result in any behavioral alterations. As expected, CENPE inhibition resulted in significant mitotic delay with increased numbers of mitotic cells with unaligned chromosomes (Fig. 3b). On average, it took CENPE inhibitor-treated cells 7–8 h to complete mitosis. This delayed mitosis due to unaligned chromosomes is a known hallmark of CENPE depletion^38,43^. Critically, when both CENPE and MEK are inhibited, we observed a slightly reduced mitotic delay compared to CENPE inhibition alone, suggesting that MEK has a minor role in generating a prolonged mitotic arrest. More importantly, a large number of cells died during mitosis, suggesting that MEK has a role in generating the anti-apoptotic signal during prolonged mitotic arrest^34^ (Fig. 3b).

To further investigate the fates of cells that completed mitosis, we utilized flow cytometry to investigate any anomalies with replication and chromosome segregation. Notably, combined treatment for 48 h results in significant accumulation of diploid (4n) and polyploid (>4n) cells (Fig. 3c, d). The cells with 4n chromosomes are either G2/M phase cells or G1 cells that failed cytokinesis and became binucleated. On the other hand, the cells with >4n chromosomes most often arise because they first fail cytokinesis and re-replicate their DNA. This process is usually prevented in non-transformed cells where P53 function is intact^44–47^. To better understand this, we used fluorescence microscopy to differentially visualize microtubules and DNA content. In line with flow cytometry analysis, we detected a considerable number of bi- and multi-nucleated cells after the combinatorial treatment (Fig. 3e, Supplementary Figure 7 a–b). These results suggest that the cells that escaped mitotic cell death under combined CENPE and MEK inhibition continue to proliferate as polyploid cells.

### Assessing drug synergy in vivo

To assess drug synergy in vivo, we designed three separate in vivo experiments. Firstly, we aimed to see if short-term combinatorial CENPE and MEK inhibition in in vivo settings recapitulate our in vitro findings in terms of mitotic phenotype. Secondly, we aimed to understand whether combinatorial treatment blocks tumor formation and, finally, whether this will also result in significant tumor volume reduction after the tumors have already formed. To understand the potential mechanism of cell death in vivo, we orthotopically implanted PDX366 tumors into the pancreas of male athymic nude mice and allowed them to grow for weeks and begun treatments of PDX tumors in vivo for 72 h and analyzed tumor cell morphologies and mitotic index in tumor cells from hematoxylin and eosin (H&E) staining. Notably, consistent with in vitro results, combined treatment with MEK and CENPE inhibition resulted in a significant increase in mitotic cells (Fig. 3f, g). To assess whether combinatorial treatment will synergistically block tumor formation, the orthotopically implanted tumors were allowed to grow for a week and mice were randomized into four drug treatment groups: control, trametinib, CENPE inhibitor, and trametinib plus CENPE inhibitor. Importantly, the volume of tumors that were treated with a combination of CENPE and MEK inhibitors were significantly smaller than any of the single treatments and were barely detectable by magnetic resonance imaging (MRI; Fig. 3h). We then wanted to know if combinatorial treatment would result in synergistic reduction of tumor volumes after the tumors reached to certain size. Thus, in an independent cohort of mice, we allowed tumors to be formed for 4 weeks and started single and combination drug treatment. Notably, in these experiments, neither of the single drug treatments resulted in significant tumor growth reduction; however, the combination of MEK and CENPE inhibitors synergistically and significantly reduced tumor volume (*p* < 0.01, *t*-test) after 4 weeks of treatment in vivo (Fig. 3i).

### Modeling drug sensitivity in cancer through CRISPR screening

**Fig. 4:**
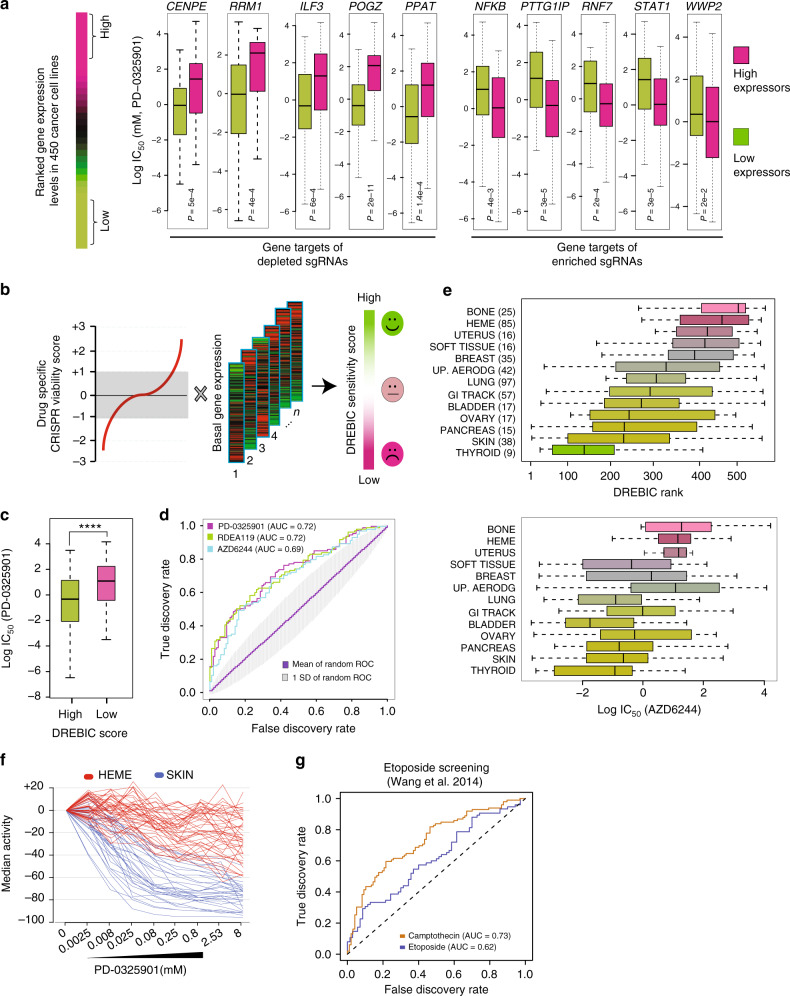
Drug response evaluation by in vivo CRISPR Screening (DREBIC) approach. **a** The 450 CGP cancer cell lines^15^ were ranked according to the expression levels of the indicated genes. Box plots depict the distribution of log IC_50_ growth inhibition values of MEK inhibitor PD-0325901 in the high- (top quartile) and low-expressing cell lines (bottom quartile). Statistical significance was calculated by Kolmogorov–Smirnov test. Comparable results were obtained for other MEK inhibitors. **b** DREBIC integrates gene-specific CRISPR viability scores with basal expression levels to model drug response phenotype. **c** Box plot showing a significant (*p* < 0.0001, Kolmogorov–Smirnov test) growth inhibition (IC_50_) difference in response to PD-0325901 MEK inhibitor in ~450 cancer cell lines scoring high (top quartile) or low (bottom quartile) in DREBIC score analysis. **d** Receiver operation characteristics curves demonstrating true vs. false discovery rate of DREBIC prediction of cellular responses to three separate MEK inhibitors (PD-0325901 red, AZD6244 blue, and RDEA119 green). The random DREBIC score distribution was generated by 10,000 permutations of the same number of genes (Supplementary Figure 12). The average ROC curve of permutation analysis is shown in purple (AUC = 0.5) and the area corresponding to one standard deviation of prediction is marked in gray. **e** The box plots in the top panel are showing the distribution of tissue-specific DREBIC scores of CGP cell lines as ordered by ascending median DREBIC score. The lower panel box plots are showing log IC_50_ growth inhibition values of AZD6244 MEK inhibitor drug for the same cancer types. **f** The PD-0325901 MEK inhibitor drug response curves are shown for hematopoietic (Heme, red) and melanoma cancer cell lines (Skin, blue) from the CCLE data set^13^. **g** The ROC curve shows the accuracy rate of etoposide-based DREBIC analysis of cellular response to three independent topoisomerase inhibitors from CPG data set. The CRISPR-gene viability scores for etoposide were obtained from a previously published in vitro CRISPR screening by Wang et al.^11^. In the box plots, bounds of the box spans from 25 to 75% percentile, center line represents median, and whiskers visualize 5 and 95% of the data points. Symbol **** denotes *P* < 0.0001

Our small molecule inhibitor studies led us to hypothesize that the endogenous expression levels of genes identified from a CRISPR screening is a key determinant of overall cellular response to MEK inhibitors. In other words, we postulated that cells with low levels of CENPE or RRM1 would be significantly more sensitive to trametinib treatment compared to cells with higher levels of these proteins. To test this, we utilized the CCLE^13^ and the CGP^15^ data sets where the responses of a large collection of cell lines have been tested against a large collection of drugs. The CGP tested 131 drugs on a panel of 624 cell lines, while the CCLE project tested 24 drugs on 947 cancer cell lines. Despite some minor inconsistencies^48,49^, together these data sets provide an unprecedented resource by delivering detailed drug sensitivity, gene expression, and mutational profiles of thousands of cancer cell lines. Although trametinib was not tested in either data set, four other MEK inhibitors (PD-0325901, AZD6244, RDEA119, and CI-1040) were tested in the CGP on 429, 408, 429, and 394 cell lines, respectively. In the CCLE, PD-0325901 and AZD6244 were each tested in ~500 cancer cell lines. To study the correlation between MEK inhibitor sensitivity and the expression levels of the gene targets of top enriched and depleted sgRNAs, we initially ranked all the cell lines according to the expression levels of the top hits from our CRISPR screen. Then, we analyzed the half-maximal inhibitory concentration (IC_50_) growth inhibition values for the MEK inhibitor in cells expressing the top and bottom quartiles of each gene. In line with our expectation, we found that cell lines that express lower levels of depleted genes, such as *CENPE, RRMI, ILF, POGZ*, and *PPAT*, are significantly more sensitive to MEK inhibitors (*p* < 0.0001, Kolmogorov–Smirnov test) (Fig. 4a). In contrast, the cells that express lower levels of genes that are enriched in the CRISPR screen, such as *PTTG1IP, RNF7, WWP2, STAT1*, and *NFKB1*, are significantly more resistant to MEK inhibitors (Fig. 4a).

These finding led us to investigate whether overall drug response can be modeled by combined actions of gene-specific CRISPR viability scores and basal expression levels. To test this hypothesis, we devised the DREBIC approach which captures the relative essentiality of drug targets (CRISPR screening viability score) and their relative abundance through their messenger RNA expression levels for a given cell type. DREBIC assigns a relative sensitivity score for each sample by simple algebraic multiplication of the gene-specific CRISPR viability score with the sample-specific gene expression level (Fig. 4b, Supplementary Figure 8, Supplementary Software 1, Supplementary Data 2–3). Therefore, once the screening is performed for a given drug, the DREBIC approach can be used to calculate cell-type-specific drug sensitivity scores as long as the gene expression profile is available.

We initially constructed cell-type-specific DREBIC scores based on CRISPR viability scores of all genes in the screen. To investigate the accuracy of DREBIC modeling of drug sensitivity, we compared it to the experimental drug response of cell lines in the CGP^15^ and CCLE datasets^13^. To be able to assess the power of DREBIC prediction, we stratified cells as responders (the top quartile) and non-responders (bottom quartile) based on the experimental drug IC_50_ response. We performed this stratification for each of the drug separately (Supplementary Figure 9). Notably, the DREBIC scores of the responders and non-responders were significantly different from each other (*p* < 0.001, *t*-test) (Fig. 4c). To better quantify the accuracy of the DREBIC prediction, we utilized receiver operating characteristic (ROC) analysis^50^ and used the area under the ROC curve (AUC) as an assessment of the prediction accuracy. Interestingly, DREBIC predicted drug sensitivity much better than random when it was constructed based on the CRISPR score of all of the genes (~4000) (Supplementary Figure 10). Additionally, we found that the combined actions of the 175 most depleted and 225 most enriched genes captures the drug response and results in the highest predictive power, further optimizing the scoring system (Fig. 4d, Supplementary Figure 11). We also performed a 10,000-permutation analysis by randomly taking the same number of enriched and depleted genes from the sgRNA-targeting gene pool. As expected, the mean AUC of random models was 0.5 with a standard deviation of 0.1, while the optimized DREBIC scored 0.73 (Fig. 4d, Supplementary Figure 12). These results indicate that DREBIC prediction power is not only better than random but is also significantly higher than the prediction power of permutated scores of DREBIC when it is constructed from the same number of randomly picked genes in the screen (*p* < 0.0001, Wilcoxon rank test). Interestingly, we noted that DREBIC has a higher prediction power when it is constructed from the in vivo screening data (AUC of 0.73 vs 0.68) (Supplementary Figure 13).

Next, we evaluated whether DREBIC could identify cancer-type-specific vulnerabilities to MEK inhibitors. We segregated CGP cancer cell lines by tissue of origin and then ranked each type by their average DREBIC scores (Fig. 4e). The analyses indicate a wide range of differences in DREBIC-based drug response of cancer types, which is in line with the known biology of these cancers. For example, pancreatic cancers and melanoma cell lines, which have aberrantly active MEK signaling due to oncogenic *KRAS* and *BRAF* mutations, were modeled and are predicted to be the most responsive. On the other hand, hematopoietic and sarcoma cell lines had the lowest DREBIC scores and thus are predicted to be the least responsive to MEK inhibitors. To further verify that DREBIC prediction rates are in line with the experimental data, we compared cancer-type-specific average DREBIC scores with the experimental log IC_50_ growth inhibition rates for the MEK inhibitors. We observed a strong trend in the consistency between the DREBIC score-based ranking and the actual experimental data, suggesting that DREBIC captures the inherent biological response of various cancer cell lines (Fig. 4e). Comparable results were obtained for other MEK inhibitors in the CGP data set. For example, as shown in the detailed dose–response curves of multiple cell lines in Fig. 4f, the hematopoietic cancer lines (HEME) are substantially more resistant to PD-0325901 compared to the melanoma (SKIN) cancer cell lines. Interestingly, our results from DREBIC analysis and from the experimental drug treatment data indicate that thyroid cancer is substantially sensitive to MEK inhibitors (even more than PDAC and melanoma). Notably, the aberrantly active RAF–RAF–MEK signaling pathway is well appreciated in thyroid cancers^51–53^ which may present an exploitable avenue for targeted treatments in this cancer.

To investigate the premise of DREBIC approach for other drugs, we analyzed previously published CRISPR screening data. Wang et al.^11^ performed CRISPR screens to identify genes implicated in cell survival under the selective pressure of a DNA topoisomerase II (TOP2A) inhibitor, etoposide. Notably, when we performed comparable analysis to Fig. 4d, we observed that etoposide-mediated CRISPR viability scores modeled the cellular response of a large compendium of cancer cells (CGP)^15^ to other topoisomerase inhibitors with high accuracy (Fig. 4g). This analysis suggests that once a CRISPR screen is performed for a given drug, the DREBIC approach can be utilized to model drug-specific cellular responses and identify relatively good responder cell types from poorly responding cancer cell lines.

### DREBIC identifies mutations that alter cellular fitness

**Fig. 5:**
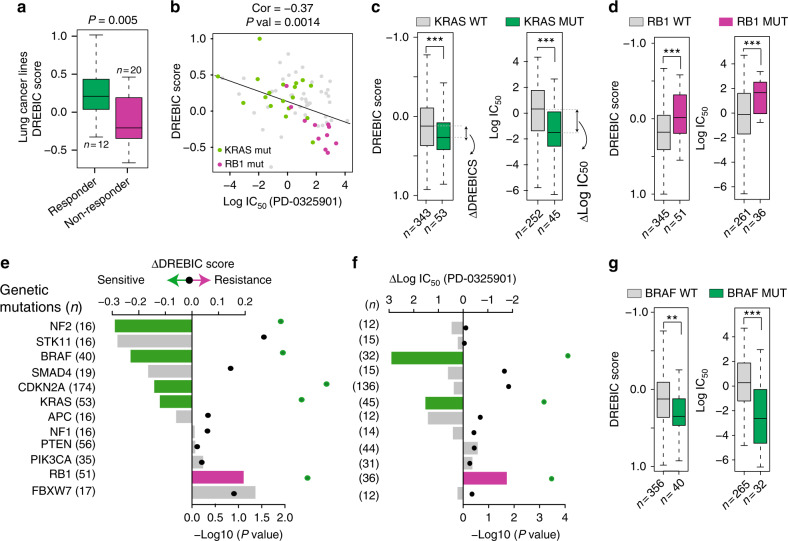
DREBIC reveals genotype-specific MEK inhibitor sensitivity. **a** Box plot showing DREBIC scores for the responder and non-responder lung cancer cell lines to MEK inhibitor (PD-0325901). **b** Scatter plot showing a correlation between DREBIC score and log IC_50_ growth inhibition values of PD-0325901 MEK inhibitor. Cell lines with *KRAS* mutations are displayed as green dots and cells with *RB1* mutations are displayed as magenta dots. **c** Box plots show the DREBIC score and log IC_50_ growth inhibition due to PD-0325901 treatment in *KRAS* WT (gray) and *KRAS* mutant (green) CGP cancer cell lines. The difference in drug response and DREBIC scores between these two groups has been defined as a difference between medians (∆DREBIC and ∆log IC_50_). **d** The same analysis as performed in (**c**) has been carried out for *RB1* WT and *RB1* mutant cells. **e** Bar plot shows the difference in DREBIC score (∆DREBIC) between WT cell lines and mutant cell lines with indicated genes. The significance of the difference was calculated by double-sided *t*-test and *p* values are shown with dots on the right side of the panel. Bars with statistically significant differences are marked with magenta and green. **f** Bar plot shows the difference in median log IC_50_ scores of PD-0325901 MEK inhibitor drug response (∆Log IC_50_) in WT cell lines and cells with mutations in the indicated genes. The significance of the difference has been calculated as in (**e**) and *p* values are shown with dots on the right side of the panel. Bars with statistically significant differences are marked with magenta for positive difference and green for the negative difference. **g** Box plots showing the DREBIC score and log IC_50_ growth inhibition due to PD-0325901 treatment in *BRAF* WT (gray) and mutant (green) CGP cell lines. Significance assessed by Kolmogorov–Smirnov test for all panels except (**b**). In the box plots, bounds of the box spans from 25 to 75% percentile, center line represents median, and whiskers visualize 5 and 95% of the data points. Significance is defined as: **p < 0.01, ***p < 0.001

Inter- and intra-tumor heterogeneity is a major challenge in predicting the tumor response to a given drug. We therefore tested to see if DREBIC could differentially score responder and non-responder cell lines within the same cancer type. To do this, we applied trametinib-based DREBIC to lung cancer cell lines. Notably, the cell lines that respond to MEK inhibition have significantly higher overall DREBIC scores compared to non-responder cell types (Fig. 5a). Furthermore, we observed a strong inverse correlation between DREBIC scores and log IC_50_ experimental growth inhibition data (Fig. 5b, *p* = 0.0014, Pearson's correlation), suggesting that DREBIC analysis can stratify heterogeneous drug response within the same cancer type. Notably, when we further stratified lung cancer cell lines into small and non-small cell lung cancer, we still observed a comparable correlation, but particularly enhanced negative correlation in small cell lung cancer (Supplementary Figure 14). To investigate potential reasons for the differential MEK inhibitor sensitivity in lung cancer, we segregated the cells according to mutational status of major cancer-driving mutations. Importantly, we found that the cells with the lowest DREBIC score have *RB1* mutations. In contrast, the MEK inhibitor-sensitive lung cancer cell lines are enriched with *KRAS* mutations (Fig. 5b), suggesting that DREBIC analysis is capturing the inherent biology of these cells as oncogenic KRAS mutant cells dependent on aberrant MEK signaling pathway. Importantly, the inverse correlation between the response of cells with *KRAS* and *RB1* mutations to MEK inhibitors was even more strongly evident when all CGP cell lines were segregated based on their mutational status and comparatively analyzed for the median DREBIC score and the median log IC_50_ growth inhibition (Fig. 5c, d).

These findings led us to test whether DREBIC analysis can identify differential vulnerabilities of other genetic mutations to MEK inhibitors. To do this, we stratified the CGP cell lines according to their mutational status and calculated the difference of the median DREBIC score (ΔDREBIC) and the difference of the median log IC_50_ growth inhibition (Δlog IC_50_) for cell lines harboring various genetic mutations. Notably, the analysis indicates that cells with *NF2*, *BRAF*, *CDKN2A*, and *KRAS* mutations are significantly more sensitive to MEK inhibitors, whereas *RB1* mutations significantly increase the cellular resistance to MEK inhibitors (Fig. 5e–g). Notably, DREBIC predicted that six of the genetic mutations would be associated with significant sensitivity to MEK inhibitors (*KRAS, CDKN2A, SMAD4, NF2, BRAF*, *and STK11*), and the actual experimental data (MEK inhibitor-mediated IC_50_ growth inhibition) recapitulated the prediction; all 6 of them were positively associated with drug sensitivity (four of them significantly, *p* < 0.05) (Fig. 5e, g). For example, DREBIC experimental data showed that *BRAF, KRAS and CDKN2A* mutant cells were significantly more sensitive to MEK inhibitors, whereas *RB1* mutant cells were significantly more resistant to MEK inhibitors. Interestingly, although not significant for experimental data (*p* > 0.05) likely due to limited cell numbers (*n* = 12), the general trend in DREBIC and experimental drug response data indicate that NF2 mutations result in increased sensitivity to MEK inhibitors. In line with this, recent reports indicate that *NF2* loss-of-function mutations promote RAS signaling and sensitize cancer cells to MEK inhibitors^54^. These results suggest that in addition to confirming known genetic vulnerabilities to MEK inhibitors such as those of *BRAF* and *KRAS* mutations^7,55,56^, DREBIC-type analysis may also be useful in predicting whether an unknown genetic mutation will positively or negatively affect overall cellular response to a given drug.

## Discussion

The CRISPR-mediated pooled loss-of-function screen can robustly identify conditional or context-dependent essential genes^24^. Here, we utilized this tool in a clinically relevant PDX model of PDAC to identify genetic targets whose deletion will positively or negatively increase overall fitness to trametinib. The framework identified multiple genes whose depletion created a conditional lethality with MEK inhibition. Our findings suggest an interesting inter-dependency of proper kinetochore function and mitogen-activated protein kinase (MAPK) signaling. The role of MAPK signaling in G1-S transition in the cell cycle is well established, but its role in G2-M transition and mitosis is relatively understudied. Interestingly, MEK1/2 and extracellular signal-related kinase-1/2 (ERK1/2) have been observed to localize to spindle poles and to the midbody during cytokinesis^57^. Furthermore, an independent study mapped CENPE as a candidate mitotic substrate for MAPK^58^. More recently, Mayes et al.^59^ used a small-scale shRNA screening against a prioritized set of genes to identify effective CENPE inhibitor drug combinations and identified MEK/ERK pathway as a major target. Our results are in line with these findings and further expand the candidate MEK inhibitor drug combinations to other kinetochore function proteins. More critically, our long-term movies suggest potential mechanism of lethality between the two seemingly unrelated pathways. Our findings show that inhibition of both MEK and CENPE results in significant cell death during mitosis, indicating that MEK signaling is required to overcome apoptotic signals during mitotic delay.

In addition to identifying synergistic drug targets, we show that results from CRISPR screenings can be exploited to predict drug response in cancer cells, which remains a formidable challenge for precision medicine. There are three major contributors to this challenge. First, not all the primary and secondary targets of small molecule drugs are known. Second, the relative importance, i.e., the level of survival fitness provided by each drug target, is hard to estimate. While gene expression changes, due to a drug treatment, can inform potential signaling pathways and gene targets of the drug, it is still difficult to know which of the differentially regulated genes are the key determinants of drug response. The final major contributor to the challenge of drug response prediction is inter- and intra-tumor heterogeneity at the level of drug targets. Basal gene expression profiles have been heavily interrogated through sophisticated machine learning and statistical computational models in an attempt to predict drug responses^13,15,60–62^. The models are typically trained on the datasets where the gene expression and drug response of a large compendium of cell lines are known and tested on a comparable data set where the basal gene expression is known but the response is unknown^61^.

The DREBIC approach we present here is a fundamentally different concept of drug response prediction. Firstly, the gene sets that contribute to the DREBIC score are based on an unbiased functional genomics data. The gene-specific CRISPR viability scores directly inform about the relative importance of each gene in contributing to the cellular fitness to the drug treatment. DREBIC-type approaches are likely to improve our ability to overcome the top two aforementioned challenges in predicting drug response. Secondly, DREBIC requires smaller set of input data. Previous methods require experimental and transcriptome data from large cohort of patient and cell lines to train the model and then used the same cohort or additional independent data to test the prediction accuracy of the trained model^61,62^. The prediction accuracy of such approaches is particularly limited when a novel drug or new drug combination is to be tested. In contrast, DREBIC does not require a large compendium of experimental drug response data. Once constructed from a screen under a drug selection, DREBIC can be applied to any cell type as long as the basal gene expression is known.

The DREBIC analysis we performed here is based on the in vivo screening of ~4000 genes. Performing more complex screenings in vivo is challenging because only limited number of cells can be injected into pancreas. However, it is reasonable to expect that whole-genome level CRISPR KO screenings may further improve the prediction power of DREBIC-type approaches. Furthermore, since the DREBIC score is an absolute but ordinal measure of drug sensitivity relative to other samples, one can expect higher prediction accuracy as the number of samples increase. We also envisage the power of DREBIC-like approaches to increase as more drug-screening data accumulate in various cells. Once the gene essentiality scores are known through screening of multiple drugs, DREBIC analysis is expected to help the precision medicine research by matching the patient’s gene expression profile with the most suitable drug or drug combinations.

## Methods

### Cell culture and drug treatments

Patient-derived tumor cells, PDX366, were maintained in RPMI-1640 medium containing 10% fetal bovine serum (FBS) and cultured in a humidified (37 °C, 5% CO_2_) incubator. This line is established from a poorly differentiated metastatic tumor with low stromal content and mutant for *KRAS*, *P53,* and *SMAD4* but WT for *P16* genes, and it was authenticated by the University of Virginia Biomedical Research Facility in 2010 as previously described^16^. PDAC cancer MPANC-96 and BxPC3 cells (ATCC, Manassas, VA) were cultured in RPMI-1640 medium supplemented with 10% FBS and %1 penicillin/streptomycin in a humidified (37 °C, 5% CO_2_) incubator. Trametinib (EuroAsia TransContinental, Mumbai, India), GSK923295 (ChemieTek, Indianapolis, IN, USA), Aurora A/B inhibitor (ZM447439, Selleckchem, cat. no. S1103), and PLK1 inhibitor (BI-2536, MedChemExpress, cat. no. HY-50698) were dissolved in sterile DMSO to make 5 mM stock solutions. Aliquots of the stock solutions were stored at −20 °C.

### MTT cell viability and cytotoxicity assay

PDX366, mPANC-96, and BxPC3 cells were seeded in a clear, flat-bottom 96-well plate (Corning) in triplicate at a density of 5 × 10^3^ cells per well. The following day, cells were treated with trametinib, GSK923295, ZM447439, and BI-2536 alone or a combination with trametinib for 72 h prior to MTT (3-(4,5-dimethylthiazolyl)-2,5-diphenyltetrazolium bromide) to determine effects of drugs on cell viability and proliferation. Culture media were replaced with fresh RPMI (phenol free) which has 10% FBS and 10% MTT (5 mg/ml) and incubated for 4 h in a humidified (37 °C, 5% CO2) incubator. Then, 100 µl MTT solvent (10% SDS in 0.01 M HCL) was added to each well and cells were incubated overnight. The absorbance was read at 595 nm. By using similar approach, IC_20_ value of trametinib was calculated in the range of 10–15 nM for in vitro screening of PDX366 cells.

### Time-lapse imaging

PDX366 cells were seeded in four-chamber slides at 1 × 10^5^ cells per well. The following day, cells were treated with CENPE inhibitor, trametinib, or a combination of CENPE inhibitor and trametinib for 24 h. The long-term time-lapse imaging was performed using Zeiss Observer Z1 wide-field microscope in a humidified environmental control chamber in the presence of 5% CO_2_ at 37 °C with continuous drug exposure for 24 h. To visualize chromosomes in live-cell imaging, 200 nM SiR-DNA (Cytoskeleton Inc.) was added to the cells 2 h before the start of time-lapse imaging. Image sequences were viewed and analyzed using ZEN lite software. For each group, cell behavior during mitosis were traced and quantified manually in an unbiased way. Movie stills were generated using Velocity software (PerkinElmer).

### Validation of KO efficiency by western Blot

After in vivo and in vitro screening, two best gRNAs were chosen for each gene for further validation. *Bsm*BI cutting site was replaced into Gecko library one plasmid system to allow us to insert our target gRNA sequence. Following gRNA sequences have been used in the validation experiment. *CENPE*-sg1: GCTGATAGGATGGCGGAGGA, *CENPE*-sg2: GAAACCATTGTAGCCTTGTA, RRM1-sg1: GTAATCCAAGGCTTGTACAG RRM1-sg2: GTCAGGGTGCTTAGTAGTCA. PDX366 cells were virally infected to express Cas9 and gRNA to produce stable cell lines. After 4 days of puromycin selection (0.5 µg/ml), lysates were prepared in RIPA buffer and equal amounts of lysates were loaded in NuPAGE 4–12% Bis-Tris gradient gel (Invitrogen cat. no. NP0335). While i-blot PVDF membrane (cat. no. IB401001) was used to transfer the RRM-1 protein, semi-dry transfer apparatus was chosen for CENPE due to its large molecular size (~300 kDa). RRM-1 (ab137114), CENPE (sc-22790), and actin (Sigma-A1978) antibodies were used in the western blot. Same stable cell lines were also used in the MTT cell viability assay.

### Calculation of drug synergism

The CI values were calculated with the Chou–Talalay method^31^ using Compusyn software according to constant ratio design between drug combinations. The fraction affected (Fa) was calculated from the single and combinational drug treatments. CI values of <1, 1, and >1 indicate synergy, additive, and antagonism between drugs, respectively.

### Generation of CRISPR-sgRNA library pool and viral infection

The lentiviral-expressing WT Cas9 plasmid under the promoter of EF1a was driven from a human gecko library plasmid after removal of the gRNA sequence. PDX366 cell lines were cultured in vitro as a heterogeneous population to keep the initial diversity of the tumor and virally infected with virus only expressing WT Cas9 were for 1 day. Next day, fresh media were added to the PDX366 cells and 0.5 µg/ml puromycin selection started for 4 days. The sgRNA libraries were kind gifts from Dr. Sabatini (MIT) and the sgRNA pool is produced according to a previously published protocol (http://www.addgene.org/static/data/08/61/acb3ad96–8db6–11e3–8f62–000c298a5150.pdf). The “nuclear” sub genomic library pool from Wang et al.^11^ has been used in this study (Addgene catalog no. 51047). This sgRNA pool is originally designed to target 3733 transcription factors, epigenetic regulators, and other nuclear function genes where each gene is targeted by ~10 different sgRNAs. In our sequencing, we identified, in total, 47,234 sgRNAs, some of which were represented by low number of reads and targeted additional genes outside the nuclear pool. Stable Cas9-expressing PDX366 cells were selected for 4 days with 5 µg/ml blasticidine with serial dilutions of a virus to find the MOI of ~0.3. Cells were harvested from 12 × 15 cm plates and combined prior to orthotopic injection into mice or in vitro screening.

### In vivo CRISPR screening

WT Cas9-expressing and sgRNA-infected PDX366 cells were injected into the pancreas of 6–7-week-old athymic nude mice (Envigo, Indianapolis, IN). Briefly, mice were anesthetized, and their left flank opened to exteriorize the pancreas. Cell lines had been harvested and resuspended for each mouse to receive 8 × 10^6^ cells in 150 µl Matrigel®Growth Factor Reduced Basement Membrane Matrix (Corning, Corning, NY). Cells were injected directly into the pancreas and the abdomen was closed in two layers. Mice that were randomized to receive trametinib (0.3 mg/kg orally, once daily; EuroAsia TransContinental) began treatment 4 days post injection. Tumors were imaged at the conclusion of the experiment using MRI (University of Virginia Molecular Imaging Core, Charlottesville, VA). After 4 weeks of growth, tumors were harvested and weighed, and samples collected for further analysis. All in vivo experiments were conducted in conjunction with University of Virginia Comparative Medicine with the approval of the Institutional Animal Care and Use Committee. Tumors were formalin fixed and submitted to the University of Virginia Research Histology Core Lab for processing and H&E staining. A board-certified pathologist who specializes in gastrointestinal cancers scored tumor sections for mitotic bodies under high magnification. For in vivo drug treatments, tumor pieces (~10 mm^3^) were orthotopically implanted onto the pancreas of 6- to 8-week-old male athymic nude mice. Tumors were allowed to grow for 4 weeks and the mice were randomized into four drug treatment groups: control, MEK inhibitor, CENPE inhibitor, and MEK inhibitor plus CENPE inhibitor. To detect potential synergistic activity, low doses of trametinib MEK inhibitor and GSK923295 CENPE inhibitor were used. Mice received single or combinatorial trametinib (0.3 mg/kg oral daily for 4 weeks) and GSK923295 (62.5 mg/kg intraperitoneal injection, 3 days on 4 days off for 2 weeks). Serial volumetric MRI was used to assess the treatment responses. This study was carried out in strict accordance with the recommendations in the Guide for the Care and Use of Laboratory Animals of the National Institutes of Health. The animal protocol was approved by the Animal Care and Use Committee of the University of Virginia (PHS Assurance #A3245–01).

### CRISPR-sgRNA library sequencing

Tumors were harvested from control and trametinib-treated mice after 4 weeks of treatment and snap frozen. Entire tumors were used to obtain genomic DNA with the following brief protocol. First, tumor samples were minced into small pieces and lysed with 8 ml SDS lysis buffer (100 mM NaCl, 50 mM Tris-Cl pH 8.1, 5 mM EDTA and 1% wt/vol SDS). Then, 100 µl proteinase K (20 mg/ml) was added to the solution followed by 55 ^o^C overnight incubation. After 2500 × *g* 10 min centrifugation, 1 ml aliquot was used for Et-OH precipitation. Pellets were resuspended in RNase-containing water. For each tumor sample, 70 µg genomic DNA was used together with outer primers for the first PCR in a total 700 µl PCR (7 × 100 µl reaction in PCR tubes). Phusion high fidelity DNA polymerase (NEB-M0530) was used for 20× cycle PCR reaction. After combining all first PCR reactions into a single tube, 10 µl of this mixture was used for the second PCR (15× cycle) with inner primer pairs in which the forward primer has a 6-bp adaptor sequence. All primer sequences are listed in Supplemental Table 2. Second PCR was run on 2% gel and the bands around 270 bp were cut and cleaned with the Qiagen gel extraction kit. Equimolar amounts of each PCR fragment were mixed and used for subsequent high-throughput sequencing with customized sequencing and indexing primers. Library was sequenced on Illumina Miseq platform to get average 5 million reads for each sample.

### CRISPR-Cas9 screening data analysis

Sequencing reads from CRISPR/Cas9 screenings were first de-multiplexed with cutadapt (v. 1.8.3). Total length 56 nt (sequencing barcode and sample barcode) were supplied to the program with the requirements that at least 36 nt of this barcode had to be present in the read so that it can be assigned to an individual tumor isolated from the PDX model. More than 99% of reads were assigned to one of the seven in vivo samples: three trametinib-treated models (further refer to as treatment), three control models (further refer to as control) and cells from the day of injection (further refer to as day 0), and three in vitro samples: day 0, treatment and control giving about 5 million reads in each sample. After de-multiplexing and removing sequencing and sample barcodes, the abundance of each sgRNA was assessed and normalized among samples with the use of MAGeCK v. 0.5.2. About 87% of reads contained correct sgRNA sequences.

Downstream data analysis was performed in RStudio v. 0.99.484 with R v. 3.3.0 following previous publications^11^ with slight modifications. We performed the following analysis to identify genes whose depletion positively or negatively altered the overall cellular fitness. The first step of this analysis was to calculate the relative abundance of each sgRNA by comparing normalized counts for each sgRNA between corresponding experiments. For example, treatment to control or treatment to day 0 sample. In case of in vivo tumors, as a control we took average of three control experiments. Resulting numbers were log-transformed giving log-fold change (LFC) of abundance for each sgRNA in each of comparisons. The second step of the analysis was to assign to each gene a CRISPR viability score, which is defined as a median of *z*-scored transformed LFC of the relative abundance of sgRNAs targeting this gene. For in vivo screen we log-transformed a median calculated for three experiments (each treatment related to average of control experiments). The significance of enrichment or depletion was calculated for each gene by Kolmogorov–Smirnov test between *z*-scores of sgRNAs targeting this gene and control sgRNA. To extract genes that are most probably drivers of resistance or sensitivity to MEK inhibition, we sought genes that present consistent enrichment or depletion of sgRNAs targeting the gene across all samples. First, we assess whether each individual sgRNA is changed in each sample by comparing its LFC with 25–75% percentile of LFC of control sgRNA. Next, we checked whether each sgRNA is consistently enriched or depleted across replicates. The sgRNAs that consistently enriched/depleted across all samples were classified as truly enriched/depleted sgRNA. Finally, we filtered the genes based on the highest number and the median LFC of the gene targeting truly enriched/depleted sgRNAs.

### Drug response stratification

We classified cancer cell lines from the CGP and CCLE data sets into three groups: responder, non-responder, and ambiguous based on their experimental drug responses (IC_50_) as depicted in Supplementary Figure 7. For each drug, the lowest quartile (25%) was stratified as responders, the highest 25% quartile was classified as non-responders. Since most drugs were tested on ~500 different cell lines, approximately 125 cell lines contributed to the responder and the same number contributed to non-responder groups for each of tested drugs.

### Construction of DREBIC approach

The DREBIC score has been constructed according to the following schematics (Supplementary Figure 6). Let gene expression data be organized into an expression matrix *G* where each column corresponds to a sample *S* to be scored and each row corresponds to a basal gene expression of *N* genes. The rows of matrix *G* are sorted by the gene-based CRISPR viability score in descending order. First, expression values are log transformed with respect to mean expression of each gene separately. Contribution to the total score from each gene is calculated by multiplying CRISPR viability score by LFC of gene expression and enriched genes are multiplied by negative 1 (−1) value. Finally, DREBIC score for each sample is calculated as a sum of contributions from each gene.

1 $${\mathrm{DREBIC}}_S = \mathop {\sum}\limits_{i = 1}^{N_D} {C_iG_{S,i}} - \mathop {\sum}\limits_{i = 1}^{N_E} {C_{N - i}G_{S,N - i}},$$

where *S* is a sample index, *N*_*E*_ and *N*_*D*_ are parameters of the model, namely number of enriched and depleted genes incorporated into the model (see section below), and *C* is a vector of the genes’ CRISPR viability scores ordered in descending order (same as rows of matrix *G*). At the end values are linearly transformed to range from –1 to +1. Above algorithm was coded in R (Supplementary Software 1, and illustrative data in: Supplementary Data 2 and 3). To measure model’s prediction accuracy, we use the AUC. ROC curves were calculated by ROCR package^63^ (v.1.0-7) in RStudio.

To further optimize DREBIC prediction accuracy and find a minimal set of genes required to obtain satisfactory prediction accuracy (measured as AUC) of DREBIC, we optimized two parameters of our model (see description above). First, we separated two parts of the model and optimized them separately obtaining an optimal number of depleted genes and an optimal number of enriched genes. We performed this analysis by calculating a series of AUCs with several enriched and depleted genes ranging from 5 to 600. Results for the PD-0325901 MEK inhibitor from the CGP data set are shown in Supplementary Figure 9. For the part of the model composed of enriched genes only, the optimal number of genes is 225, while for the other part composed of depleted genes only the number is 175 genes. Obtained numbers of genes are consistent among all four MEK inhibitors.

To assess the significance of CRISPR viability scores’ contribution to prediction power, we performed permutation validation. Specifically, we picked two random sets of 175 genes and 225 genes to calculate random DREBIC score and repeated these 10,000 times. Each time AUC was calculated as a measure of the accuracy of prediction. Results are plotted as cumulative distribution functions in Supplementary Figure 10. The *p* values have been calculated as a fraction of cases that gave more extreme AUC than our model.

We calculate the average ROC curve as follows. First, for each individual curve at each point on the *x*-axis, we took the maximal value of *y*. The average of those maximal values was taken as the average ROC curve *y* value at each *x*. The standard deviation of an average ROC curve was calculated as standard deviation of those maximal y values. Average values of random AUC are 0.5 and standard deviations are around 0.1.

### Antibodies

The antibodies (Abs) used in this study were used at the following dilutions. First Ab: CENPE (rabbit) 1:500 in 3% milk; RRM-1 (rabbit) 1:1000 in 3% milk; actin (mouse) 1:2000 in 3% milk. Second Ab: anti-mouse 1:10,000 in 3% milk; anti-rabbit 1:10,000 in 3% milk.

### Drug response due to genetic mutations

To perform genotypic study presented on Fig. 5e, first we picked genes that are mutated in at least 12 cell lines. Next, for each of the gene from the list we calculated median DREBIC score and median IC_50_ (as shown on the box plots in Fig. 5c) for mutants and wild type. Finally, we presented data on bar plots. Significance has been assessed with Kolmogorov–Smirnov test by comparing WT and mutant DREBIC scores and IC_50_ respectively.

## Electronic supplementary material

Supplementary Information

Description of Additional Supplementary Files

Supplementary Data 1

Supplementary Data 2

Supplementary Data 3

Supplementary Software

## Data Availability

Data set generated within this study is attached as Supplementary Data 1. Data sets from Cancer Genome Project can be retrieved at: drug sensitivity data are Supplementary Data 1 in Garnet at al. paper (https://www.nature.com/nature/journal/v483/n7391/extref/nature11005-s2.zip), and expression data are deposited on ArrayExpress under the accession number E-MTAB-783. Data sets from Cancer Cell Line Encyclopedia can be obtained at CCLE project portal (https://portals.broadinstitute.org/ccle) after free registration. Data set from Wang et al. was published as Supplementary Table 2 (http://science.sciencemag.org/highwire/filestream/594960/field_highwire_adjunct_files/1/1246981s2.xlsx). Sequencing data are available from (SRA) under Project ID SUB4477193; bioproject PRJNA488636 (https://submit.ncbi.nlm.nih.gov/subs/biosample/SUB4477193/). The individual accession codes are as follows: SAMN09938197: ControlTumor1, SAMN09938198: ControlTumor2, SAMN09938199: ControlTumor3, SAMN09938200: TreatedTumor1, SAMN09938201: TreatedTumor2, SAMN09938202: TreatedTumor3, SAMN09938203: Day 0, SAMN09938204: DMSO, SAMN09938205: Treated.
