# Supplementary material for: CRISPR knockout screening identifies combinatorial drug targets in pancreatic cancer and models cellular drug response
Source: Nat Commun. 2018 Oct 15;9:4275. doi: 10.1038/s41467-018-06676-2 (PMC6189038; doi:10.1038/s41467-018-06676-2)
Supplement: Supplementary file 1 — Supplementary Information [file 41467_2018_6676_MOESM1_ESM.pdf]

Supplementary Figures and Table cover page

Szlachta et al. ***CRISPR knockout screening identifies combinatorial drug targets in pancreatic cancer and models cellular drug response***

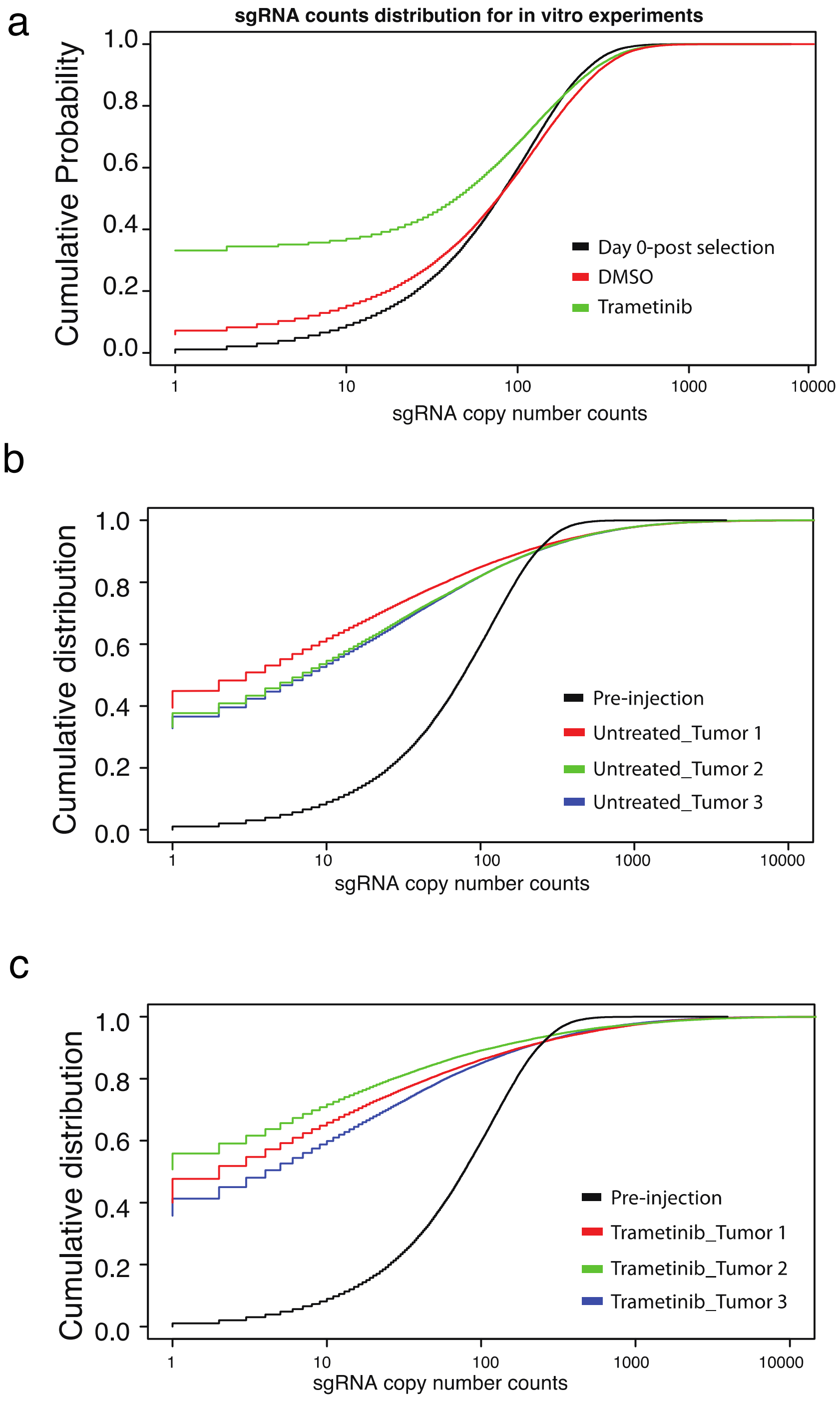

Supplementary Figure 1: Cumulative distributions of counts of detectable sgRNAs in A) in vitro screen; B) three control treated tumors and C) three trametinib treated tumors from in vivo screening.

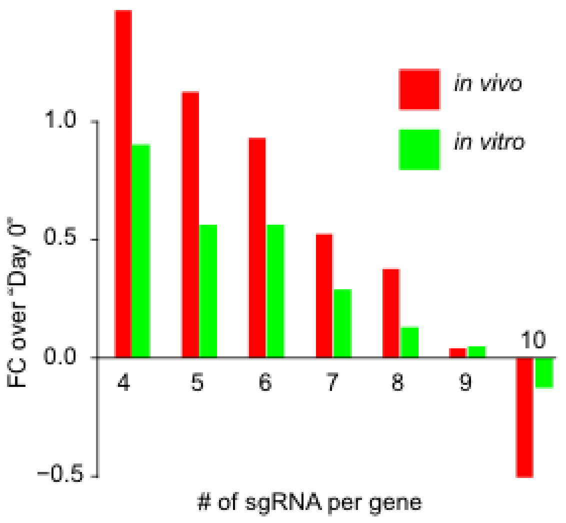

Supplementary Figure 2: Bar plot presentation of global depletion of sgRNAs. For each gene, we calculated the number of sgRNAs that could be detected in "Day 0" and control samples of both in vivo and in vitro experiment. Next we counted the number of genes that are targeted by the indicated number of sgRNAs (x-axis) in each experiments. Y-axis (bar height) represents fold change in a number of genes targeted by a given number of sgRNAs compared to Day 0. Positive FC value indicates that the total number sgRNAs targeting a gene is generally depleted both in vitro and in vivo screening. The figure highlights that at each cut off value, more sgRNAs are depleted in *in vivo* settings compared to *in vitro* setting.

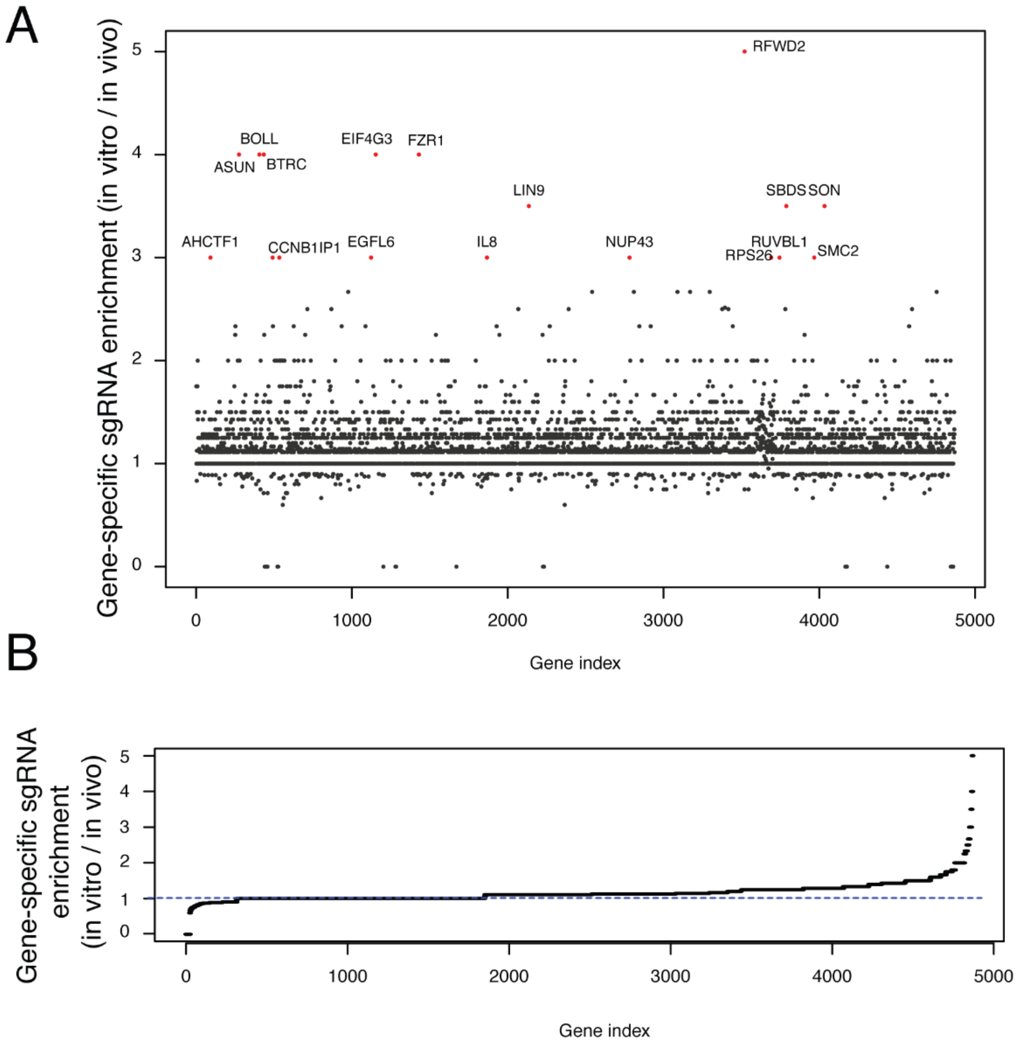

Supplementary Figure 3: Comparative analysis of *in vitro* and *in vivo* screens. **a** Each dot represents ratio of a number of sgRNAs (with more than 3 counts) between *in vitro* and *in vivo* screens for a single gene. Genes are ordered alphabetically. Enrichment score of 1 indicate no change between the number sgRNAs for a given gene in *in vitro* and *in vivo* screening. Most genes have *in vitro/in vivo* score of more than 1, indicating that sgRNAs are globally depleted/underrepresented *in vivo.* The genes that seem to more dramatically depleted *in vivo* are named. **b** The figure presented in A is ranked ordered to highlight that the enrichment score for most genes is above 1, which is displayed as dotted line.

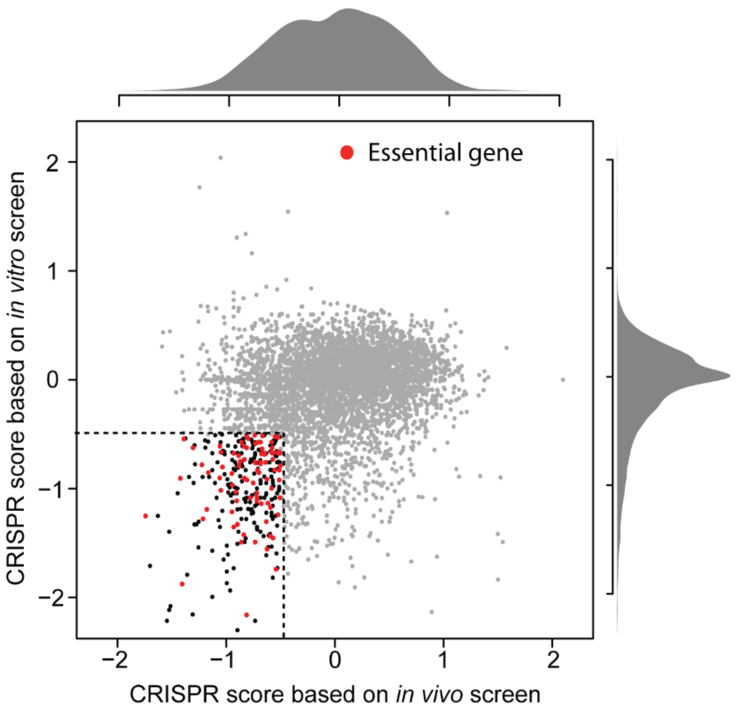

Supplementary Figure 4: Comparative analysis of CRISPR viability scores for *in vivo* and *in vitro* control experiments. Central panel (scatter plot) presents CRISPR z-scores (control treatment relative to "day 0") for *in vitro* (y axis) and *in vitro* (x axis) screens. Side panels present kernel density estimation of corresponding z-score, which indicate that in vivo screening is associated with wider z-score distributions compared to in vitro screening. The lower guardant of the figure highlights the genes depleted both in vitro and in vivo control samples. The genes, which are previously identified as essential fitness genes by *Hart et al., (Cell*, 2015; PMID: 26627737) are displayed as red dots. Tart et al identified approximately 1600 genes, which is ~8 % of the genome. Among the ~4000 genes in our screening, which are enriched for important transcription factors and epigenetic regulators, ~15% of them are the essential fitness genes (2X enrichment/genome). Notably, among the genes that are commonly depleted both in vitro and in vivo samples, more than 30% are fitness genes, which is 4X enrichment compared to genomic control.

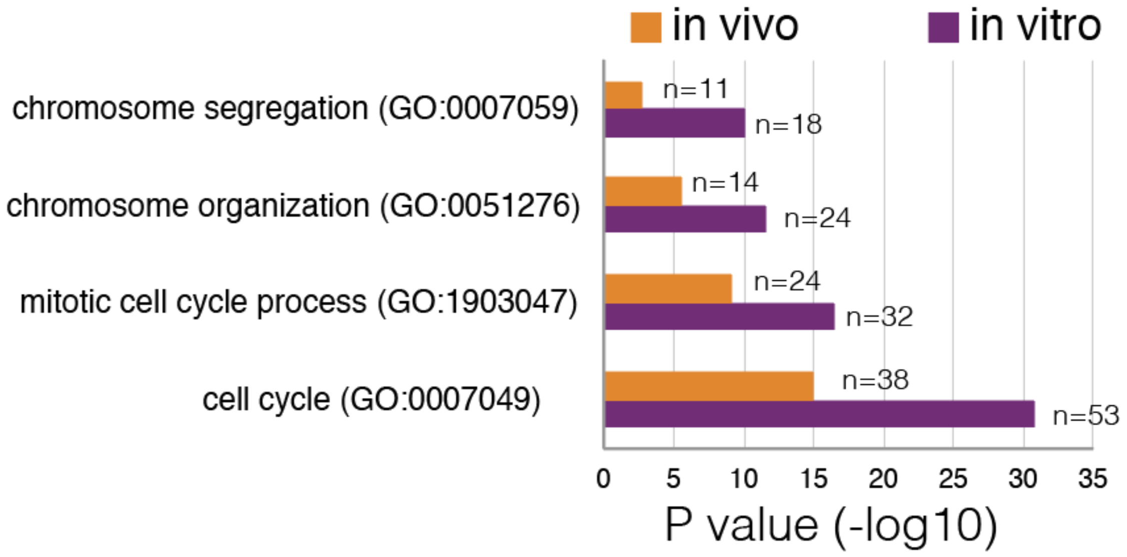

Supplementary Figure 5: Gene Ontology enrichemnt analysis for top 100 genes depleted in treatment vs control comparison in *in vivo* and *in vitro* screening. Number of genes in each category are indicated next the respective bar graphs.

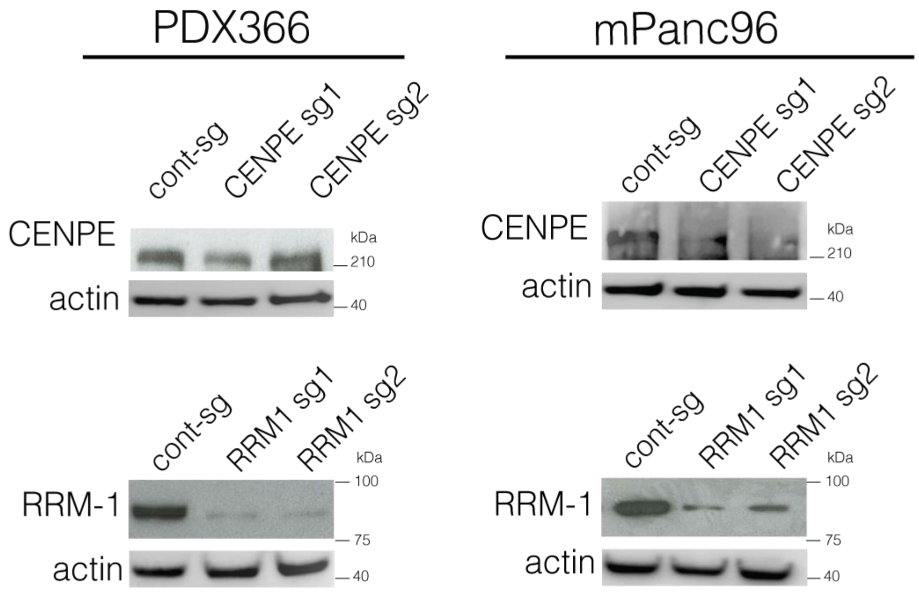

Supplementary Figure 6: Western blots shows the amount of CENPE and RRM1 protein in control and targeted sgRNAs in two different pancreatic cancer cells by using two best gRNAs according to the CRISPR viability score. β-actin is used as a loading control**.**

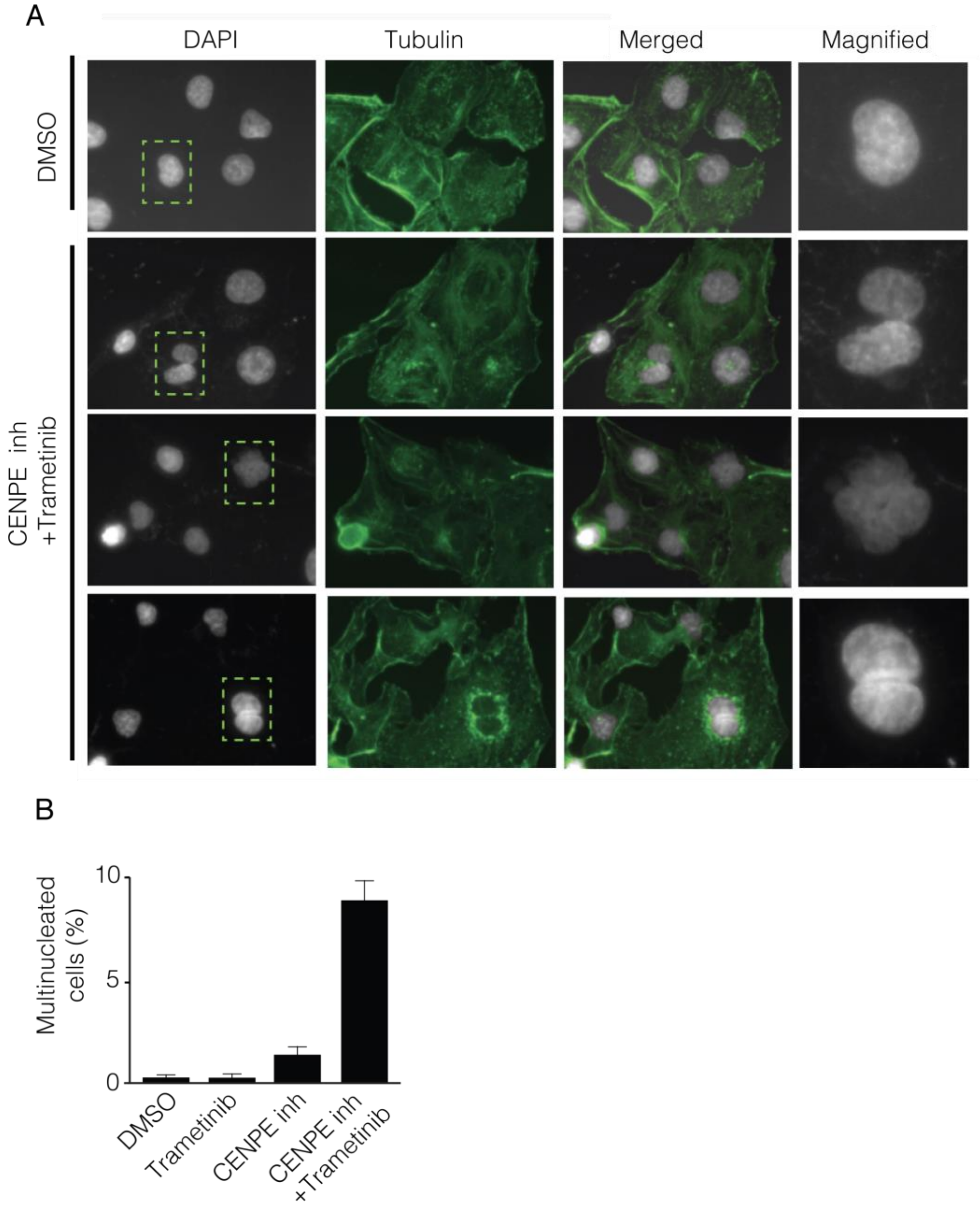

Supplementary Figure 7: Immunofluorescent images of PDX366 PDAC cells treated with control and combinatorial (Trametinib and CENPE inhibitors) are shown after DAPI and tubulin staining in the first two left columns respectively. Third column indicates the merged images. Last column belongs to magnified images of the multi-nucleated cell which is shown by the green dotted boxes in the DAPI staining. **b** Bar graphs represent percent of multinucleated cells after 24 hours of control, single-agent, or combined treatments.

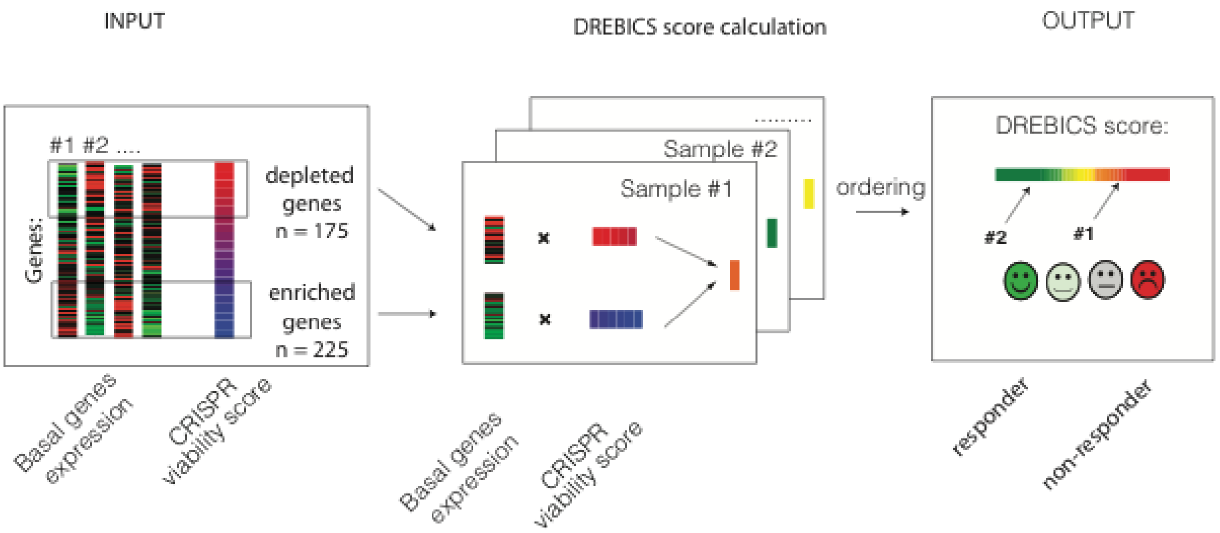

Supplementary Figure 8: Figure shows the outline of DREBICS algorithm (for mathematical formulation of algorithm see materials and methods section). For each sample, basal gene expressions are combined with CRISPR viability scores for most depleted and enriched genes. This operation is repeated for all of the samples. At the end, the total results are rank-sorted according to the DREBICS score. The ranked scores are then compared to experimental data sets for prediction accuracy.

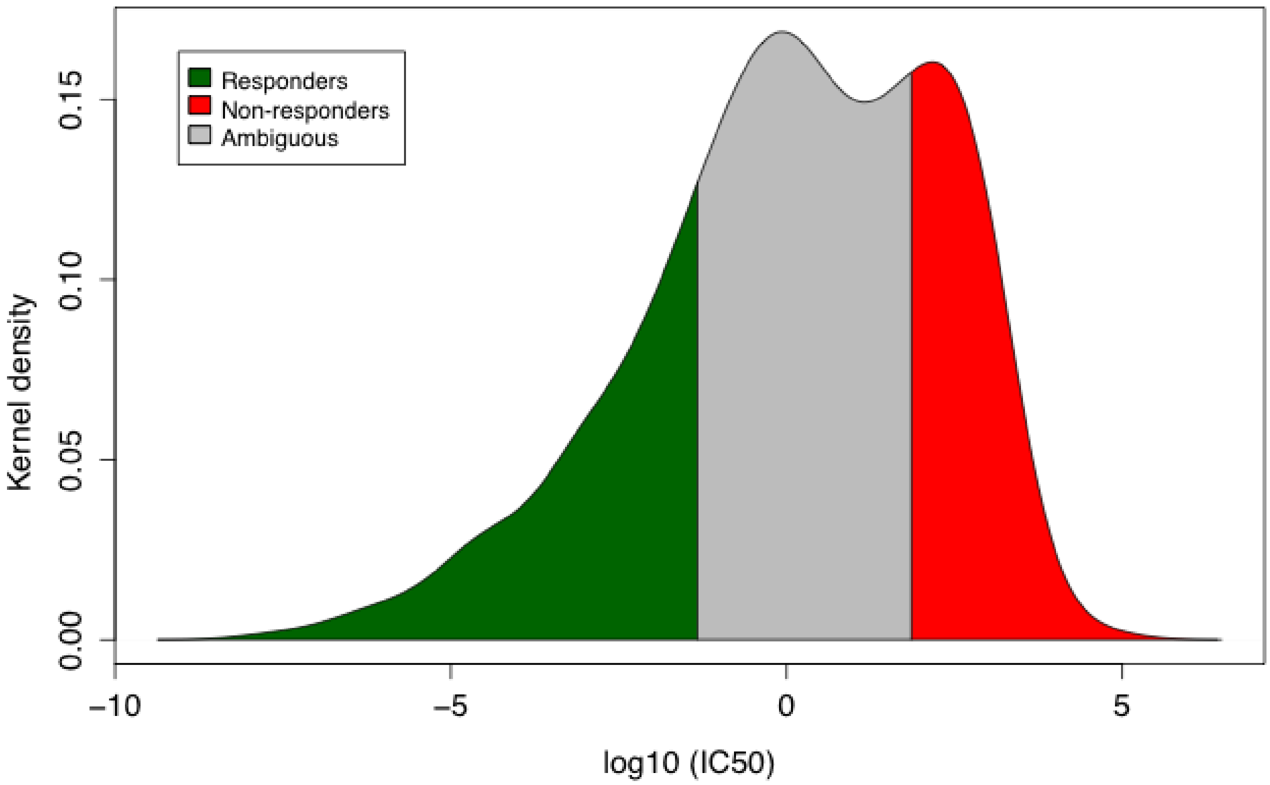

Supplementary Figure 9: Classifying cancer cell lines as drug responders and non-responders based on IC_{50} growth inhibition. The graph is showing the kernel density plot of the distribution of IC_{50} values for 429 cell lines which sensitivity to PD-0325901 MEK inhibitor was tested in CGP. The lowest quartile was stratified as responders, and highest as non-responders. Remaining cells as well as not tested against PD-0325901 were denoted as ambiguous.

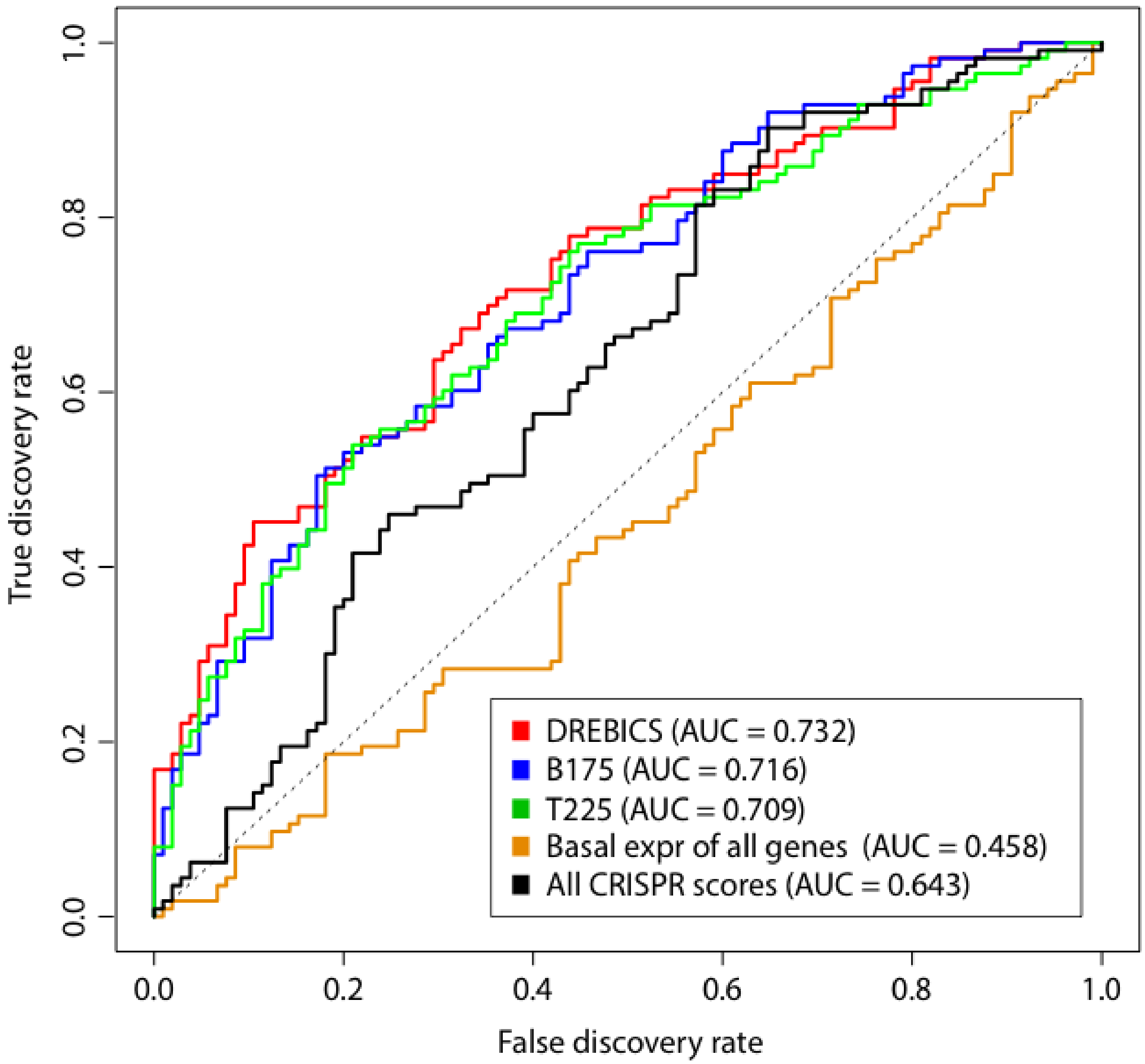

Supplementary Figure 10: Receiver operating characteristic curves (ROC) of optimal model (DREBIC, red line) and sub-optimal models. DREBIC is performing better when it constructed from top 225 enriched genes and bottom 175 depleted genes (area under ROC curve is 0.732) than when it is constructed from either of these set of genes. B175 and T225 represent the score when DREBIC is constructed from bottom 175 depleted genes and top 225 enriched genes, respectively. Model established on basal gene expression only (orange) or with all CRISPR scores (black) are only slightly better than random models (one standard deviation from the random models based on permutation analysis), areas under ROCs are 0.458 and 0.643, respectively.

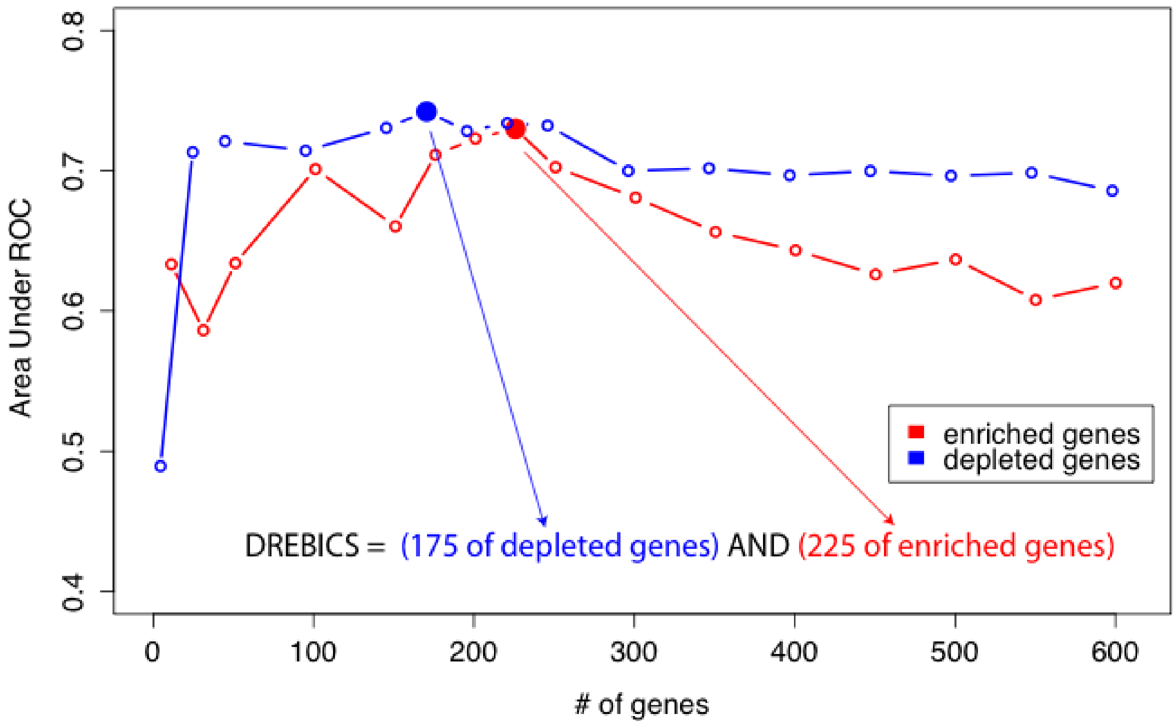

Supplementary Figure 11: Prediction accuracy of DREBICS components (enriched and depleted genes separately) based on the various numbers of top depleted and enriched genes. The best prediction accuracy is obtained when 225 enriched genes (red dot) and 175 depleted genes (blue dot) are used.

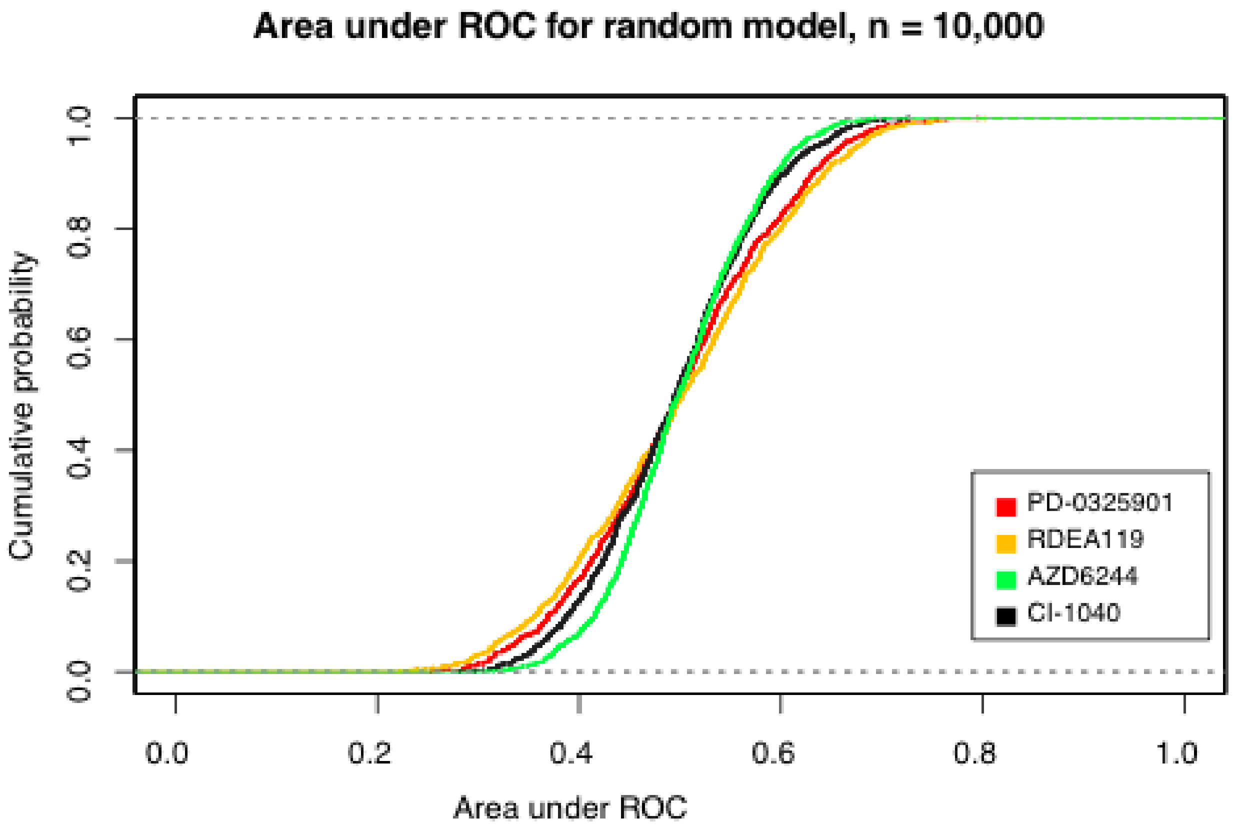

Supplementary Figure 12: Cumulative distribution of AUC scores of 10,000 permutations using random 175 depleted genes and 225 enriched genes.

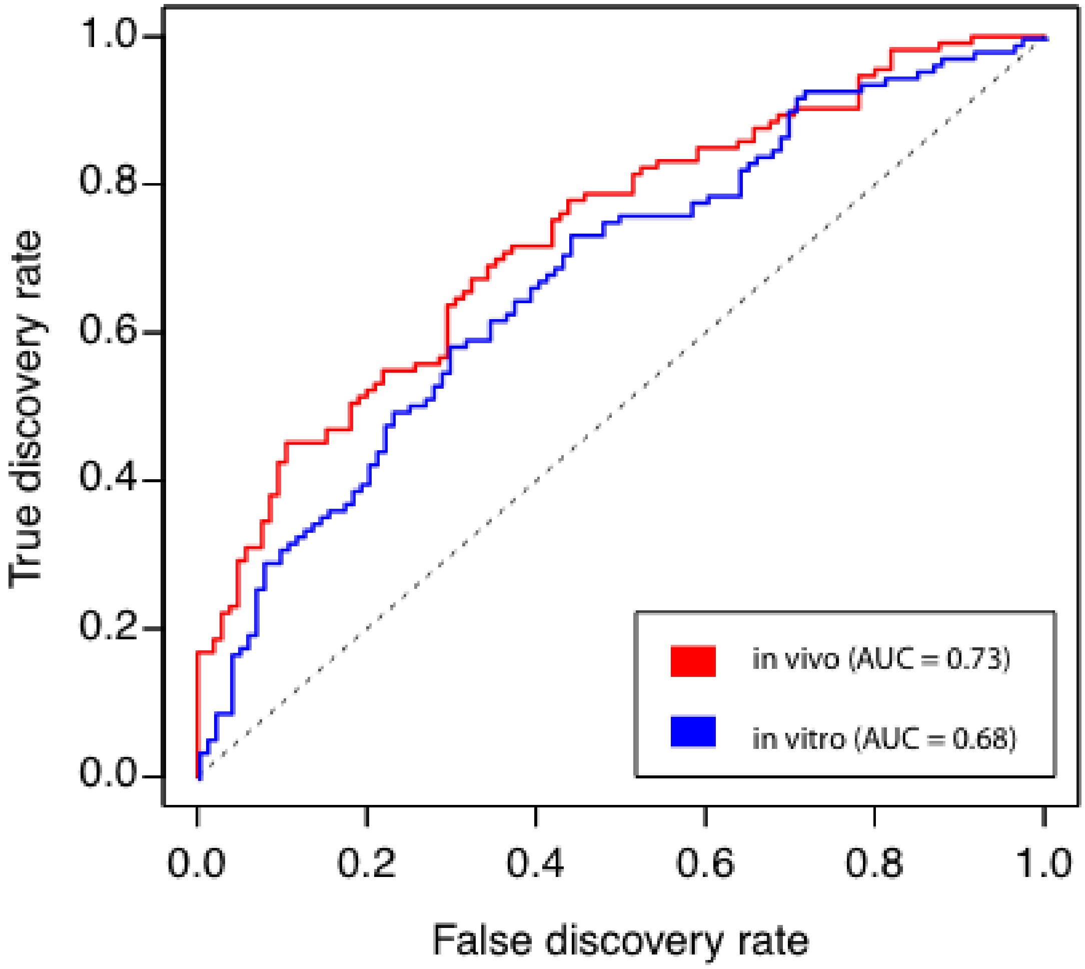

Supplementary Figure 13: Receiver operating characteristic curves (ROC) of optimal model (DREBICS) based on *in vivo* (red) and *in vitro* (blue) screening results. DREBICS is performing better with *in vivo* CRISPR z-scores (Area under ROC curve: 0.73 vs 0.68 respectively) in terms of predicting cellular response to MEK inhibitor PD-0325901.

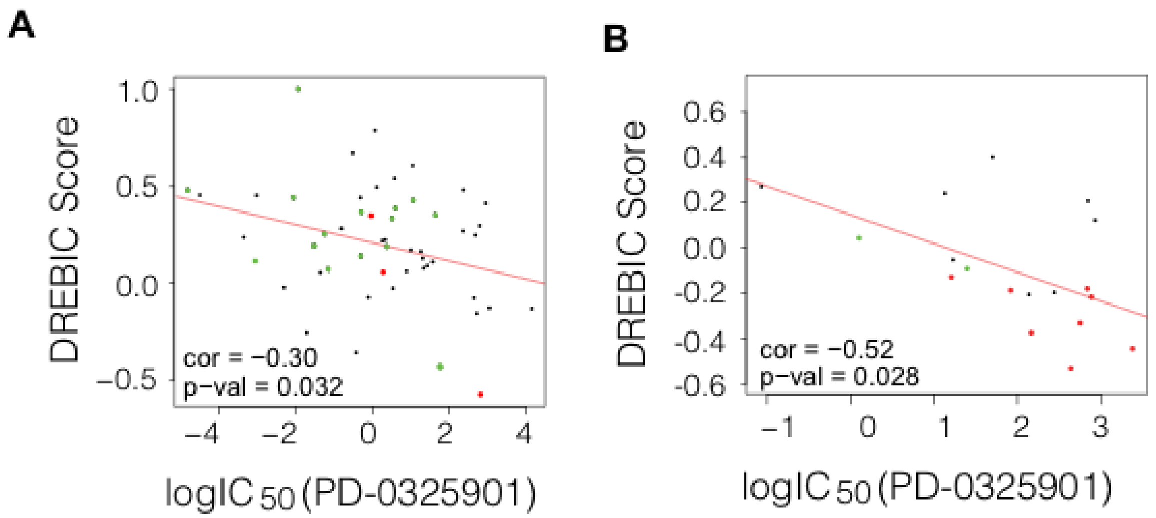

Supplementary Figure 14: Scatter plot showing a correlation between DREBIC score and log IC50 growth inhibition values of PD-0325901 MEK inhibitor in lung cancer cell lines: **a)** non small cell lung cancer, and **b)** small cell lung cancer. Cell lines with KRAS mutations are displayed as green dots and cells with RB1 mutations are displayed as red dots.

**Table 1**	**in vivo**						**in vitro**		
	Untreated			Trametinib			Day 0	DMSO	Trametinib
	Tumor 1	Tumor 2	Tumor 3	Tumor 1	Tumor 2	Tumor 3			
sgRNAs with > 1 counts	28621 (60.6%)	31744 (67.2%)	31564 (66.8%)	30348 (64.3%)	28342 (60.0%)	23273 (49.2%)	47233 (100%)	44426 (94%)	31575 (66.9%)

Supplementary Table 1: Number of sgRNAs detected after various in vitro and in vivo selections.

Primer names	Primer sequence	index sequence	Sample name	Treatment
Outer-F	GCCGGCTCGAGTGTACAAAA			
Outer-R	AGCGCTAGCTAATGCCAACTT			
lib-IN-F-index1	CAAGCAGAAGACGGCATACGAGATCatcacgTTTCTTGGGTAGTTTGCAGTTTT	atcacg	day0	
lib-IN-F-index4	CAAGCAGAAGACGGCATACGAGATCtgaccaTTTCTTGGGTAGTTTGCAGTTTT	tgacca	Tumor 1	Trametinib
lib-IN-F-index5	CAAGCAGAAGACGGCATACGAGATCacagtgTTTCTTGGGTAGTTTGCAGTTTT	acagtg	Tumor 2	
lib-IN-F-index6	CAAGCAGAAGACGGCATACGAGATCgccaatTTTCTTGGGTAGTTTGCAGTTTT	gccaat	Tumor 3	
lib-IN-F-index7	CAAGCAGAAGACGGCATACGAGATCcagatcTTTCTTGGGTAGTTTGCAGTTTT	cagatc	Control 1	Control
lib-IN-F-index8	CAAGCAGAAGACGGCATACGAGATCacttgaTTTCTTGGGTAGTTTGCAGTTTT	acttga	Control 2	
lib-IN-F-index9	CAAGCAGAAGACGGCATACGAGATCgatcagTTTCTTGGGTAGTTTGCAGTTTT	gatcag	Control 3	
lib-IN-F-index2	CAAGCAGAAGACGGCATACGAGATCcgatgtTTTCTTGGGTAGTTTGCAGTTTT	cgatgt	in-vitro	Control
lib-IN-F-index3	CAAGCAGAAGACGGCATACGAGATCttaggcTTTCTTGGGTAGTTTGCAGTTTT	ttaggc	in-vitro	Trametinib
lib-IN-Rev	AATGATACGGCGACCACCGAGATCTACACCGACTCGGTGCCACTTTT			
custom seq primer	CGGTGCCACTTTTTCAAGTTGATAACGGACTAGCCTTATTTTAACTTGCTATTTCTAGCTCTAAAAC			
custom indexing primer	TTTCAAGTTACGGTAAGCATATGATAGTCCATTTTAAAACATAATTTTAAAACTGCAAACTACCCAAGAAA			

Supplementary Table 2: Names and sequences of primers for PCR-amplicon DNA library preparation
