# Supplementary material for: CRISPR knockout screening identifies combinatorial drug targets in pancreatic cancer and models cellular drug response
Source: Nat Commun. 2018 Oct 15;9:4275. doi: 10.1038/s41467-018-06676-2 (PMC6189038; doi:10.1038/s41467-018-06676-2)
Supplement: Supplementary file 2 — Description of Additional Supplementary Files [file 41467_2018_6676_MOESM2_ESM.pdf]

File Name: Supplementary Data 1
Description: sgRNA counts in control and trametinib treatment experiments, both in vivo and in vitro.

File Name: Supplementary Data 2
Description: CRISPR scores obtained from in vivo experiments.

File Name: Supplementary Data 3
Description: Gene expressions for cell lines in CGP data set. Attached for readers

File Name: Supplementary Software 1
Description: Implementation of DREBIC algorithm in R.
